# The Application of Pearls in Traditional Medicine of China and Their Chemical Constituents, Pharmacology, Toxicology, and Clinical Research

**DOI:** 10.3389/fphar.2022.893229

**Published:** 2022-08-23

**Authors:** Yinglian Song, Wanyue Chen, Ke Fu, Zhang Wang

**Affiliations:** ^1^ College of Pharmacy, Chengdu University of Traditional Chinese Medicine, Chengdu, China; ^2^ State Key Laboratory of Southwestern Chinese Medicine Resources, Chengdu University of Traditional Chinese Medicine, Chengdu, China; ^3^ College of Ethnomedicine, Chengdu University of Traditional Chinese Medicine, Chengdu, China

**Keywords:** pearls, traditional medicine of China, chemical constituents, pharmacology, toxicology, clinical application

## Abstract

Although pearls are well known by most people, their medicinal value has not been popularized. This article collates the medicinal history of pearls over 2,000 years in China, including the application of pearls in the traditional medicine of China and their various preparations, as well as the progress of their chemical constituents, pharmacology, toxicology, and clinical research. Pearls from three different sources are used as medical materiel by 9 nationalities and 251 prescription preparations in China. In addition, pearls contain various inorganic constituents, such as calcium carbonate, trace elements, and water, and organic constituents, such as amino acids. In terms of pharmacology, pearls have many effects such as calming, improving cognitive ability, being anti-epileptic, promoting bone growth and regeneration, promoting the proliferation and migration of human microvascular endothelial cells, protecting the heart, anti-hemolysis, and anti-oxidation. In terms of toxicology, pearls are safe to take for a long time without exerting obvious adverse reactions. In terms of clinical application, pearls have been used to treat many diseases and conditions, such as convulsions, epilepsy, palpitations, eye diseases, ulcer diseases, skin diseases, or skin lesions. This article provides a reference for the application and research of pearls in the future.

## Graphical Abstract

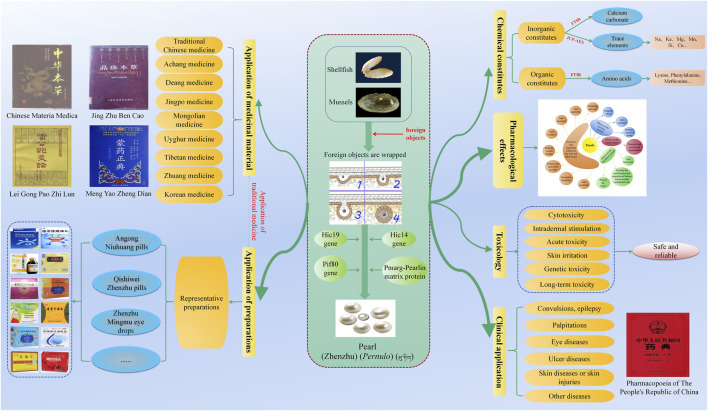

## 1 Introduction

Pearls are produced by the natural biomineralization process (Bourrat et al., 2012). Biomineralization, the biological process by which an organism produces mineralized tissue (Montagnani et al., 2011), results from a unique biological synergy (Arnaud-Haond et al., 2007). The process of pearl formation, whether by natural means or human intervention, is a response to mantle tissue damage (Cartwright et al., 2013). The formation of pearls under natural conditions rarely occurs (Wang et al., 2009; Liu et al., 2012) and is generally cultivated by artificial means. When pearl shellfish or mussels are stimulated or stressed by foreign objects or external forces, their defensive function is stimulated, and the upper mantle envelops the foreign objects, resulting in pearl formation. The part of the cells of a single layer epithelial tissue is invaginated, constantly secreting nacre on foreign objects and gradually forming a pearl sac. The pearl sac continues to secrete nacre, surrounds it, and finally forms a pearl (Koji, 1999; Ma N. et al., 2011). Pearl formation includes two consecutive stages. One is the irregular CaCO_3_ deposition on the bare nucleus; the second is that the deposition of CaCO_3_ becomes more and more regular until a mature nacre is formed on the nucleus, similar to the regeneration of shells (Sato and Komaru, 2019). Pearl formation takes about 2–3 years and is related to the expression and release of a variety of genes and proteins, such as the Hic14 gene (Jin et al., 2019), Hic19 gene (Jin et al., 2019), Pif80 gene (Zhang et al., 2018), Pmarg-Pearlin matrix protein (Bourrat et al., 2012), and pearl shell matrix protein gene (Cartwright et al., 2013). These genes and proteins jointly regulate the formation of pearls, such as their growth, shape, and size. Pearls are mainly composed of the pearl nucleus, amorphous matrix layer, and nacre layer; amongst them, the nacre layer includes the prismatic layer and aragonite layer (Wan et al., 2014; Wan, 2016; Wu, 2017). The pearl nucleus is a small ball after grinding and polishing (Ouyang et al., 2012), which can be formed naturally or artificially implanted. The amorphous matrix layer is an organic matter that adheres to the surface of the pearl nucleus or a mixture of organic matter and inorganic crystal formation (Wan et al., 2014; Wan, 2016; Wu, 2017), and its thickness affects the quality of pearls. The amorphous matrix layer is thinner or even absent in high-quality pearls. The prismatic layer is also called the calcite crystal layer, slightly different from the view that it appears only in inferior pearls and hardly in high-quality pearls. On the contrary, the prismatic layer is universally present in pearls, and its role in pearls can be summarized as regulating spatial orientation by controlling the size and shape of the pearls (Wan et al., 2014). The aragonite layer is the main constituent of pearls as it directly determines their quality (Wan, 2016). Ouyang et al. (2012) described the structure of pearls according to the classification of nucleated and non-nucleated pearls (Ouyang et al., 2012).

Pearls (Latin name: *Pernulo*, Chinese name and its Pinyin: 珍珠Zhenzhu, Tibetan name: 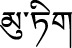) are a mineral medicine used in the traditional medicine of China. It can be obtained from bivalves such as *Pteria martensii* Dunker, *Hyriopsis cumingii* Lea, or *Cristaria plicata* Leach; it can tranquilize and quiet the spirit, improve eyesight and remove nebula, detoxify and promote granulation, and moisturize the skin and remove speckle (Committee, 2020). It has a spherical, oblong, oval, or rod shape; has a characteristic colorful sheen; and is qualitatively hard (Committee, 2020). Pearls have been used for medicinal purposes in China for more than 2,000 years (Zhang X. et al., 2014). A clear record of pearls’ efficacy has been reported as early as the Jin dynasty (AD 266–420) in *Bao Pu Zi* and *Zhou Hou Bei Ji Fang* of Ge Hong (Lu et al., 1991), indicating that pearls had been used as medicine before, and its effects were summarized by later ages. Pearls can be divided into many types according to different ways, such as natural and artificial pearls with different genesis (Zhao, 1992). Seawater and freshwater pearls have different ecological environments, and white and black pearls have different colors (Feng et al., 2005). Most of the freshwater pearls have been used in medicine; they are mostly produced amongst Chinese rivers, and China accounts for 80% of the world’s freshwater pearls production. However, most of the common pearls in the market are seawater pearls, which are often used for decoration because of their natural environmental effects, bright color, and large round integuments, but there are also many freshwater pearls in the market. Of course, natural seawater contains more organic substances and nutrients than freshwater, so seawater pearls are better for medicinal purposes (Lin et al., 2007). Pearls as medicine are generally developed in the form of pearl powder by physical grinding and used as a raw material in traditional Chinese medicine and cosmetics (Liu et al., 2018). Pearl powder makes the constituents more bioavailable because of its small particle size and large contact area, which facilitates the release of active components and absorption by the human body (Gao et al., 2008). Some studies have shown (Chen L. et al., 2019) that the particle size of pearl powder will affect its curative effect, from which nano-pearl powder is derived, and the size of pearl powder after nanometerization is smaller, the released protein is increased, and the activity is stronger. Pearls have also been used in various compound preparations due to their good bioactivity and effect, such as Qishiwei Zhenzhu pills, Ershiwuwei Zhenzhu pills, Mengyao Zhenzhu pills, Luhui Zhenzhu capsules, Zhenzhufen capsules, Zhenzhu Shaoshang ointments, Zhenzhu ointments, and Zhenzhu pulvis, which tranquilize and quiet the spirit (Sun and Qiu, 2012), remove decay and promote granulation (Xiang and Zheng, 2010), and exert whitening effect (Xiao et al., 2015).

Pearls are not only traditional natural medicine but also a kind of marine shellfish. Marine shellfish have long been used to treat inflammation, burns, scalds, wounds, cuts, and pain healing, among others (Yang, 2020). In addition, the effects of pearls on calming (Pan et al., 1999; Zhang et al., 2016), improving eyesight (Lu et al., 1990), and whitening (Yang et al., 2016a) are also significant. It has been used as a medicine in China for thousands of years and has been used by many ethnic groups in China at the same time (Zhang X. et al., 2014). In modern research, pearls or pearls combined with other medicine have also been applied to epilepsy (Zhong et al., 2010), eye diseases (Niu and Xu, 2005), and skin diseases (Xie, 2013), among others.

With the progress of medical technology and the expansion of people’s perception of traditional Chinese medicine, pearls play an increasingly important role in medical treatment. Thus, this study will further promote the use of pearls and provide a reference for future research by expounding on pearls’ application in the traditional medicine of China and their chemical constituents, pharmacology, toxicology, and clinical research.

## 2 Application of Pearls in Traditional Medicine of China

### 2.1 Records of Pearls in Traditional Medicinal Works of China

The application of pearls in China has a long history and is collected in numerous medicinal works (see Table 1 for details). The different names of pearls are real bead, mussel bead, Zhenzhuzi, medicinal bead, bead, and Lianzhu (Medicine, 1999). The application of pearls is officially recorded in Ge Hong’s *Zhou Hou Bei Ji Fang* in the Jin dynasty (AD 266–420) and Lei Xiao’s *Lei Gong Pao Zhi Lun* in the Southern and Northern dynasty (AD 420–589) (Xu, 2019). Tibetan medical works such as *Jing Zhu Ben Cao* (AD 1835) also recorded varieties of pearls (Dimaer, 1986). In terms of medicinal use, Yao Xing Lun (AD 907–960) said, “It can treat cataracts in the eyes and also down bear [*sic*] phlegm.” Hai Yao Ben Cao (AD 907–960) said that pearls “mainly improve eyesight, remove snoring, and stop diarrhea,” and a brief explanation was also given on the compatibility medicine of pearls. Ri Hua Zi Ben Cao (AD 907–960) said that they “soothe the nerves, improve eyesight, and maintain beauty.” The soothing effect of pearls was mentioned for the first time, indicating that our ancestors had a more comprehensive understanding of the main functions of pearls at this time, such as calming the mind, soothing the nerves, and improving eyesight (Medicine, 1999; Zhang and Peng, 2015; Xu, 2019). Ancient books such as Hai Yao Ben Cao (AD 907–960) and Tu Jing Ben Cao (AD 1061) also recorded the origin place and collection of pearls in detail. *Ben Cao Yan Yi* (AD 1116) said that pearls are also used to treat “convulsions and fevers in children.” During the Ming (AD 1368–1,644) and Qing dynasties (AD 1636–1912), pearls have new progress in medicinal use, especially in the aspects of astringing sores and promoting granulation due to the outstanding progress of the navigation industry. For example, Ben Cao Gang Mu (AD 1578) said that they “soothe the nerves, stop spermatorrhea and leucorrhea, relieve acne and furunculosis, treat dystocia, and remove stillbirth.” Ben Cao Hui Yan (AD 1624) said that they “calm the heart, stabilize the will, calm the soul, detoxify, remove malignant sores, and astringe internal ulcers.” Ben Jing Feng Yuan (AD 1695) recorded that “calcined ashes are used to promote granulation,” which is used to treat “burns.” Ben Cao Cong Xin (AD 1757) believed that pearls had the effects of “detoxifying, astringing sores, and promoting granulation.” At present, Materia Medica’s records on pearls’ functions have gradually improved (Zhang and Peng, 2015).

**TABLE 1:** Records of pearls in traditional medicinal works of China.

No.	Traditional medicinal works of China	Years	Records of pearls	References
1	Zhou Hou Bei Ji Fang	AD 266–420	Officially collected	Xu (2019)
2	Lei Gong Pao Zhi Lun	AD 420–589	Officially collected	Xu (2019)
3	Yao Xing Lun	AD 907–960	Treat cataracts in the eyes and depress phlegm	Medicine (1999); Xu (2019); Zhang and Peng (2015)
4	Hai Yao Ben Cao	AD 907–960	Mainly improve eyesight, remove snoring, and stop diarrhea; recorded the place of origin and collection of pearls in detail	Medicine (1999); Xu (2019); Zhang and Peng (2015)
5	Ri Hua Zi Ben Cao	AD 907–960	Soothe the nerves, improve eyesight, and maintain beauty	Medicine (1999); Xu (2019); Zhang and Peng (2015)
6	Tu Jing Ben Cao	AD 1061	Recorded the place of origin and collection of pearls in detail	Zhang and Peng (2015)
7	Ben Cao Yan Yi	AD 1116	Treat convulsions and fevers in children	Zhang and Peng (2015)
8	Ben Cao Gang Mu	AD 1578	Soothe the nerves, stop spermatorrhea and leucorrhea, relieve acne and furunculosis, treat dystocia, and remove stillbirth	Zhang and Peng (2015)
9	Ben Cao Hui Yan	AD 1624	Calm the heart, stabilize the will, calm the soul, detoxify, remove malignant sores, and astringe internal ulcers	Zhang and Peng (2015)
10	Ben Jing Feng Yuan	AD 1695	Calcined ashes are used to promote granulation	Zhang and Peng (2015)
11	Ben Cao Cong Xin	AD 1757	Detoxify, astringe sores, and promote granulation	Zhang and Peng (2015)
12	Jing Zhu Ben Cao	AD 1835	Various varieties of pearls	Dimaer (1986)

### 2.2 Application of Pearls as Medicinal Material

Pearls are derived from bivalves such as *Pteria martensii* Dunker, *Hyriopsis cumingii* Lea, or *Cristaria plicata* Leach, which are used in traditional Chinese medicine and eight ethnic groups (Achang, Deang, Jingpo, Mongolian, Uyghur, Tibetan, Zhuang, and Korean) in China (Table 2) (Jia and Zhang, 2016; Committee, 2020). In traditional Chinese medicine and ethnomedicine, pearls are mainly used to treat nervous system diseases, sores, poisoning, red eyes and nebula disorder, and skin pigmentation with good results. Amongst them, traditional Chinese medicine is mostly used to treat palpitations, eye diseases, ulcers, skin diseases, and other conditions; Achang, Deang, and Jingpo medicines are mostly used to treat convulsive seizures and ulcers; Mongolian and Tibetan medicines are mostly used to treat stroke (brain) and hemiplegia; Uyghur and Zhuang medicines are mainly used to treat insomnia with palpitations and convulsion epilepsy; and Korean medicine has special application, for nausea, diarrhea, phlegm, and other diseases.

**TABLE 2:** The application of pearls in the traditional medicine of China.

Varieties of pearls	Traditional medicine of China	Application
*Pteria martensii* Dunker	Traditional Chinese medicine	Palpitations and insomnia, convulsions and epilepsy, red eyes and nebula disorder, sores not converging, skin pigmentation
	Achang medicine	Pediatric convulsions; external use: sore throat, erosion, ulcer not close for a long time
	Deang medicine	Pediatric convulsions; external use: sore throat, erosion, ulcer not close for a long time
	Jingpo medicine	Pediatric convulsions; external use: sore throat, erosion, ulcer not close for a long time
	Mongolian medicine	*Baimai* diseases, *Sa* disease, skull injury, gout, travel pain symptom, sores, dizziness, coma, skewing of the mouth and eyes, delirious, slurred speech, numbness of limbs, hemiplegia, various poisoning symptoms, trauma, rheumatism
	Uyghur medicine	Palpitations and insomnia, diarrhea, red eyes and nebula disorder, bleeding from hemorrhoids, menorrhagia, heart flustered, neuropathy, panic disorder, ocular trauma and eyes with weak eyesight, bleeding gums, increased leucorrhea, bloody dysentery continues, spermatorrhea with premature ejaculation, measles, smallpox, various types of spots
	Tibetan medicine	Cerebral concussion, head injury, *Baimai* diseases, nebula disorder, numbness of limbs, poisoning symptoms, pediatric convulsive epilepsy, traumatic brain injury, dysphoria, chest tightness
	Zhuang medicine	Insomnia, convulsions, epilepsy, sore mouth and tongue, sore throat, sores not close for a long time
*Hyriopsis cumingii* Lea	Traditional Chinese medicine	Palpitations and insomnia, convulsions and epilepsy, red eyes and nebula disorder, sores not converging, skin pigmentation
	Korean medicine	Nausea, retention of phlegm and retained fluid, stop diarrhea, retching counterflow
	Mongolian medicine	*Baimai* diseases, *Sa* disease, skull injury, gout, travel pain symptom, sores
	Uyghur medicine	Palpitations and insomnia, diarrhea, red eyes and nebula disorder, bleeding from hemorrhoids, menorrhagia, heart flustered, neuropathy, panic disorder, ocular trauma and eyes with weak eyesight, bleeding gums, increased leucorrhea, continued bloody dysentery, spermatorrhea with premature ejaculation, measles, smallpox, various types of spots
	Tibetan medicine	Neuropathic disorders, traumatic brain injury, pediatric convulsive epilepsy, dysphoria, poisoning symptoms, cerebral concussion, chest tightness
	Zhuang medicine	Insomnia, convulsions, epilepsy, sore mouth and tongue, sore throat, sores not close for a long time
*Cristaria plicata* Leach	Traditional Chinese medicine	Palpitations and insomnia, convulsions and epilepsy, red eyes and nebula disorder, sores not converging, skin pigmentation
	Mongolian medicine	*Baimai* diseases, *Sa* disease, skull injury, gout, travel pain symptom, dizziness, coma, skewing of the mouth and eyes, delirious, slurred speech, spasms of limbs, hemiplegia, various poisoning symptoms, trauma, sores, rheumatism
	Uyghur medicine	Palpitations and insomnia, diarrhea, red eyes and nebula disorder, bleeding from hemorrhoids, menorrhagia, heart flustered, neuropathy, panic disorder, ocular trauma and eyes with weak eyesight, bleeding gums, increased leucorrhea, continued bloody dysentery, spermatorrhea with premature ejaculation, measles, smallpox, various types of spots
	Tibetan medicine	Traumatic brain injury, pediatric panic dysphoria, brain leakage (the cerebral marrow flows out with the nasal discharge, a kind of rhinitis), food poisoning, neuropathic disorders, poisoning symptoms, cerebral concussion, chest tightness, pediatric convulsions
	Zhuang medicine	Insomnia, convulsions, epilepsy, sore mouth and tongue, sore throat, sores not close for a long time

Note: the above information is from the *Dictionary of Chinese Ethnic Medicine* (Committee, 2020) and *Chinese Pharmacopoeia* (Jia and Zhang, 2016).

### 2.3 Application and Statistical Analysis of Pearls in Traditional Medicinal Preparations of China

According to statistics, pearls are used in 251 preparations, including 63 pills, 51 capsules, 40 tablets, 50 pulvis, 9 oral liquids, 16 ointments, 6 granules, 6 eye drops, 4 suppositories, and 1 each of other dosage forms (e.g., effervescent tablets, lozenges, mixtures, aerosols, and oils). A total of 227, 18, 5, and 2 preparations containing pearls are used in Chinese, Tibetan, Mongolian, and Uyghur medicines, respectively. Table 3 only displays five representative preparations in each dosage form (including Chinese, Tibetan, Mongolian, and Uyghur medicine), and all are displayed when the number of preparations is less than five. The table contains the dosage form, the name of the preparation, the traditional (ethnic) medicine it belongs to, the composition of medicinal materials, pearls dosage, and indications.

**TABLE 3:** Application of pearls in representative preparations.

Dosage forms	Name of preparations	Traditional (ethnic) medicine	Composition of medicinal materials	Dose of pearls	Indications	Possible effects of pearls
**Pills**	Angong Niuhuang pills	Traditional Chinese medicine	Gallstones of *Bos taurus domesticus*, Horn of *Bubalus bubalis*’s concentrated powder, secretions in male sachets of *Moschus berezovskii* or *Moschus sifanicus* or *Moschus moschiferus*, pearl, Cinnabar, realgar, Rhizomes of *Coptis chinensis* or *Coptis deltoidea* or *Coptis teeta*, root of *Scutellaria baicalensis*, fruits of *Gardenia jasminoides*, root of *Curcuma wenyujin* or *Curcuma longa* or *Curcuma kwangsiensis* or *Curcuma phaeocaulis*, crystallization of extracts of *Blumea balsamifera* or *Cinnamomum camphora*	15 g	Pyreticosis, evil into the pericardium, febrile convulsion, delirium, stroke coma, encephalitis, meningitis, toxic encephalopathy	Quiet the spirit
	Annao pills	Traditional Chinese medicine	Artificial gallstones of *Bos taurus domesticus*, Porcine bile (gallbladder) powder, Cinnabar, crystallization of extracts of *Blumea balsamifera* or *Cinnamomum camphora*, horn of *Bubalus bubalis*’s concentrated powder, pearl, root of *Scutellaria baicalensis*, fruits of *Gardenia jasminoides*, Realgar, root of *Curcuma wenyujin* or *Curcuma longa* or *Curcuma kwangsiensis* or *Curcuma phaeocaulis*, gypsum fibrosum, ocher, shells of *Hyriopsis cumingii* or *Cristaria plicata* or *Pteria martensii*, extracts of stems and leaves of *Mentha haplocalyx* (l-menthol)	50 g	High fever and dizziness, dysphoria and delirious speech, convulsive spasms, stroke, headache vertigo. Hypertension and all acute inflammation accompanied by persisting high fever and coma of mind	Quiet the spirit
	Qishiwei Zhenzhu pills	Tibetan medicine	Pearl, heartwood of *Santalum album*, heartwood of *Dalbergia odorifera*, Nine ocular stones (a mineral gem), stigma of *Crocus sativus*, gallstones of *Bos taurus domesticus*, artificial secretions in male sachets of *Moschus berezovskii* or *Moschus sifanicus* or *Moschus moschiferus*, etc. 70 kinds	——	*Heibaimai* diseases, *Longxue* disorder; stroke, paralysis, hemiplegia, epilepsy, cerebral hemorrhage, concussion, heart disease, hypertension and neurological disorders	*——*
	Renqing Changjue	Tibetan medicine	Pearl, cinnabar, heartwood of *Santalum album*, heartwood of *Dalbergia odorifera*, fruits of *Terminalia chebula* or *Terminalia tomentella*, gallstones of *Bos taurus domesticus*, secretions in male sachets of *Moschus berezovskii* or *Moschus sifanicus* or *Moschus moschiferus*, stigma of *Crocus sativus*, etc.	——	*Long*, *Chiba*, *Peigen* diseases, old gastroenteritis, ulcer, *Mubu* diseases, atrophic gastritis, various poisonings, syphilis, leprosy, old pyreticosis, anthrax, furuncle pain, *Ganhuangshui* (*Huangshui* diseases, that is exudates from lesions such as skin eczema, scabies, and furuncle caused by damp heat), suppuration, etc.	Detoxify and anti-ulcer
	Zhachong Shisanwei pills	Mongolian medicine	Fruits of *Terminalia chebula* or *Terminalia tomentella*, roots of *Aconitum kusnezoffii* (concocted), rhizomes of *Acorus tatarinoxjuii*, roots of *Aucklandia lappa*, artificial secretions in male sachets of *Moschus berezovskii* or *Moschus sifanicus* or *Moschus moschiferus*, *Sarcostemma acidum*, pearl, bud of *Eugenia caryophyllata*, seed kernel of *Myristica fragrans*, wood of *Aquilaria sinensis*, Limonitum, Magnetite, roots and rhizomes of *Glycyrrhiza uralensis* or *Glycyrrhiza inflata* or *Glycyrrhiza glabra*	——	Hemiplegia, paralysis, skewing of the mouth and eyes, numbness in limbs, weak waist and legs, slurred speech, arthralgia and myalgia, nerve paralysis, rheumatism, joints pain	Tranquilize and quiet the spirit
**Capsules**	Luhui Zhenzhu capsules	Traditional Chinese medicine	Concentrated dry matter of leaves juice of *Aloe barbadensis* or *Aloe ferox*, root of *Aucklandia lappa*, pearl	——	Constipation, difficulty in defecation, abdominal distension and fullness, bitter and dry mouth, *etc*. caused by Qi stagnation and heat excessive, functional constipation with the above-mentioned syndromes	Moisten intestines and health care
	Meihua Dianshe capsules	Traditional Chinese medicine	Artificial gallstones of *Bos taurus domesticus*, pearl, secretions in male sachets of *Moschus berezovskii* or *Moschus sifanicus* or *Moschus moschiferus*, secretions of *Bufo bufo gargarizans* or *Bufo melanostictus* (concocted), gallbladder of *Ursus thibetanus* or *Ursus arctos*, realgar, Cinnabar, Borax, seeds of *Descurainia sophia* or *Lepidium apetalum*, resin of *Boswellia carterii* or *Boswellia bhaw-dajiana* (concocted), resin of *Commiphora myrrha* or *Commiphora molmol* (concocted), resin of fruits of *Daemonorops draco*, wood of *Aquilaria sinensis*, crystallization of extracts of *Blumea balsamifera* or *Cinnamomum camphora*	9 g	Sore swelling and pain at the beginning, throat and gum swelling and pain, sore mouth and tongue, the above syndromes all caused by fire evil thrives in the body	Anti-inflammatory and anti-ulcer
	Ershiwuwei Shanhu capsules	Tibetan medicine	*Sarcostemma acidum*, fruits of *Terminalia chebula* or *Terminalia tomentella*, wood of *Saussurea costus*, root and rhizomes of *Glycyrrhiza uralensis* or *Glycyrrhiza inflata* or *Glycyrrhiza glabra*, bud of *Eugenia caryophyllata*, Drgonsbones (fossils of mammalian bones), flowers of *Carthamus tinctorius*, roots of *Aconitum pendulum*, pearl, artificial secretions in male sachets of *Moschus berezovskii* or *Moschus sifanicus* or *Moschus moschiferus*, cinnabar, *etc*.	——	“*Baimai*” diseases, delirious, numbness of the body, light headedness, brain pain, irregular blood pressure, headache, epilepsy and various neuropathic pains	Tranquilize
	Zhenlong Xingnao capsules	Tibetan medicine	Pearl, dried mass of exudate of *Bambusa textilis* or *Schizostachyum chinense*, stigma of *Crocus sativus*, bud of *Eugenia caryophyllata*, seed kernel of *Myristica fragrans*, fruits of *Amomum kravanh* or *Amomum compactum*, fruits of *Amomum tsao-ko*, heartwood of *Santalum album*, wood of *Aquilaria sinensis*, fruits of *Terminalia chebula* or *Terminalia tomentella*, fruits of *Terminalia bellirica*, fruits of *Phyllanthus emblic*a, roots of *Aucklandia lappa*, Bark of *Cinnamomum cassia*, fruit clusters of *Piper longum*, *Eriocheir sinensis* or *Eriocheir japonicus*, Lapis Micae *Aureus*, fruits of *Cuminum cyminum*, artificial gallstones of *Bos taurus domesticus*, artificial secretions in male sachets of *Moschus berezovskii* or *Moschus sifanicus* or *Moschus moschiferus*, fruits of *Choerospondias axillaris*, leaves of *Rhododendron anthopogonoides*, whole plant of *Corydalis impatiens*, whole plant of *Lagotis brachystachya*, Ferrous powder, fruits of *Malva verticillata*, roots and rhizomes of *Glycyrrhiza uralensis* or *Glycyrrhiza inflata* or *Glycyrrhiza glabra*, seeds of *Nigella glandulifera*	——	Stroke caused by phlegm-stasis blocking the collaterals, difficult sluggish speech, hemiplegia, skewing of the mouth and eyes	——
	Jingtian Quban capsules	Tibetan medicine	Root and rhizomes of *Rhodiola crenulata*, fruits of *Lycium barbarum*, roots of *Astragalus membranaceus* or *Astragalus membranaceus*, roots of *Angelica sinensis*, roots of *Polygonum multiflorum* (concocted), flowers of *Carthamus tinctorius*, pearl, flowers of *Rhododendron simsii*	——	Chloasma and acne caused by Qi stagnation and blood stasis	Moisturize the skin and remove speckle
**Tablets**	Fufang Zhenzhu Anchuang tablets	Traditional Chinese medicine	Nacre powder, powder of horn of *Saiga tatarica*, horn of *Bubalus bubalis*’s concentrated powder, roots of *Glehnia littoralis*, roots of *Paeonia lactiflora* or *Paeonia veitchii*, roots of *Scutellaria baicalensis*	3 g	Acne, skin eczema, dermatitis	Moisturize the skin and remove speckle
	Houjiling tablets	Traditional Chinese medicine	Artificial gallstones of *Bos taurus domesticus*, roots of *Isatis indigotica*, roots and rhizomes of *Sophora tonkinensis*, roots of *Platycodon grandiflorum*, fruits of *Terminalia chebula* or *Terminalia tomentella*, roots and root bark of *Wikstroemia indica*, roots of *Trichosanthes kirilowii* or *Trichosanthes rosthornii*, fruits of *Forsythia suspensa*, crystallization of extracts of *Blumea balsamifera* or *Cinnamomum camphora*, nacre powder, roots and rhizomes of *Achyranthes bidentata* or *Achyranthes longifolia* or *Achyranthes aspera* or *Achyranthes indica*, sterile fruits of *Gleditsia sinensis*	56 g	Mumps, tonsillitis, acute pharyngitis, acute attack of chronic pharyngitis, sore throat	Anti-inflammatory
	Ershiwuwei Zhenzhu tablets	Tibetan medicine	Pearl, seed kernel of *Myristica fragrans*, Travertine, fruits of *Amomum tsao-ko*, bud of *Eugenia caryophyllata*, heartwood of *Dalbergia odorifera*, heartwood of *Santalum album*, fruits of *Phyllanthus emblic*a, horn of *Bubalus bubalis*, gallstones of *Bos taurus domesticus in vitro* cultivation, artificial secretions in male sachets of *Moschus berezovskii* or *Moschus sifanicus* or *Moschus moschiferus*, *etc*. 20 kinds	——	Stroke, hemiplegia, skewing of the mouth and eyes, unconscious, delirious, delirium manic, *etc*.	Tranquilize
	Jingtian Quban tablets	Tibetan medicine	Roots and rhizomes of *Rhodiola crenulata*, fruits of *Lycium barbarum*, roots of *Astragalus membranaceus* or *Astragalus membranaceus*, roots of *Angelica sinensis*, roots of *Polygonum multiflorum* (concocted), flowers of *Carthamus tinctorius*, pearl, flowers of *Rhododendron simsii*	——	Chloasma and acne caused by Qi stagnation and blood stasis	Moisturize the skin and remove speckle
	Jianxin Hemier Gaozi Bananbire tablets	Uyghur medicine	Leaves of *Rumex dentatus*, flowers of *Rumex dentatus*, intestinal secretions of *Physeter catodon* or *Physeter macrocephalus*, pearl, amber, silkworm cocoons, *Sarcostemma acidum*, heartwood of *Santalum album*, gold leaf, silver foil, ruby, flowers of *Salix caprea*, whole plant of *Dracocephalum moldavica*, bud of *Rosa rugosa*	——	Palpitations, insomnia, and dreaminess	Quiet the spirit
**Pulvis**	Babao Jingfeng pulvis	Traditional Chinese medicine	Tubers of *Gastrodia elata*, dried mass of exudate of *Bambusa textilis* or *Schizostachyum chinense*, *Buthus martensii*, stems and branches of *Uncaria rhynchophylla* or *Uncaria macrophylla* or *Uncaria hirsuta* or *Uncaria sinensis* or *Uncaria sessilifructus*, artificial gallstones of *Bos taurus domesticus*, secretions in male sachets of *Moschus berezovskii* or *Moschus sifanicus* or *Moschus moschiferus*, fruits of *Gardenia jasminoides*, Lapis Micae Aureus, pearl, wood of *Aquilaria sinensis*, crystallization of extracts of *Blumea balsamifera* or *Cinnamomum camphora*, roots of *Saposhnikovia divaricata*, *etc*. 19 kinds	50 g	Infantile convulsions, fever and cough, vomiting of phlegm-drool	Tranquilize
	Fufang Zhenzhu pulvis	Traditional Chinese medicine	Shells of *Haliotis diversicolor* or *Haliotis discus hannai* or *Haliotis ovina* or *Haliotis ruber* or *Haliotis asinina* or *Haliotis laevigata* (Calcined), *Drgonsbones* (fossils of mammalian bones), kaolinite (calcined), gypsum fibrosum (calcined), pearl, artificial secretions in male sachets of *Moschus berezovskii* or *Moschus sifanicus* or *Moschus moschiferus*, crystallization of extracts of *Blumea balsamifera* or *Cinnamomum camphora*	7.5 g	Ulcer caused by heat evil accumulation, symptoms can be seen, sores are fresh and decayed	Anti-ulcer
	Shuangliao Houfeng pulvis	Traditional Chinese medicine	Pearl, srtificial gallstones of *Bos taurus domesticus*, crystallization of extracts of *Blumea balsamifera* or *Cinnamomum camphora*, rhizomes of *Coptis chinensis* or *Coptis deltoidea* or *Coptis teeta*, roots and rhizomes of *Sophora tonkinensis*, roots and rhizomes of *Glycyrrhiza uralensis* or *Glycyrrhiza inflata* or *Glycyrrhiza glabra*, powder, clumps or granules made from leaves or stems and leaves of *Baphicacanthus cusia* or *Polygonum tinctorium* or *Isatis indigotica*, Depositum urinae hominis (calcined), gypsum rubrum	——	Swollen and painful throat and gums caused by excessive heat evil in the lungs and stomach	Anti-inflammatory
	Zhuhuang pulvis	Traditional Chinese medicine	Pearl, gallstones of *Bos taurus domesticus*	500 g	Pharyngitis, tonsillitis, aptha, gangrenous stomatitis, *etc*.; throat is red and swollen; sore throat is obvious, especially when swallowing; tonsils is red and swollen, with secretions on the surface, significant pain; gangrenous stomatitis is accompanied by red and swollen ulcer, and the sores not close for a long time. Acute pharyngitis, acute tonsillitis, recurrent oral ulcer, gingivitis	Anti-ulcer
	Tumuxiang Shiweitang pulvis	Mongolian medicine	Roots of *Inula helenium*, seeds of *Momordica cochinchinensis* (calcined), *Drgonsbones* (fossils of mammalian bones) (calcined), whole plant of *Parnassia palustris*, fruits of *Terminalia chebula* or *Terminalia tomentella*, roots of *Sophora flavescens*, bud of *Rosa rugosa*, pearl (concocted with milk), rhizomes of *Zingiber officinale*, concentrated dry matter of leaves juice of *Aloe barbadensis* or *Aloe ferox* (concocted)	——	Febrile headache, headache caused by blood heat, rhinitis, toothache	Tranquilize and quiet the spirit
**Oral liquids**	Azhen Yangxue oral liquids	Traditional Chinese medicine	Pearl, solid glue made of skin or fresh skin of *Equus asinus*, roots of *Angelica sinensis*, roots of *Rehjnannia* *glutinosa* (concocted), rhizomes of *Ligusticum chuanxiong*, roots of *Paeonia lactiflora*, roots of *Astragalus membranaceus* or *Astragalus membranaceus*, roots of *Codonopsis pilosula* or *Codonopsis pilosula* or *Codonopsis tangshen*, fruits of *Ziziphus jujuba*, fruits of *Ligustrum lucidum*, rhizomes of *Polygonatum odoratum*	——	Dizziness, palpitations, skin dryness, and unhealthy skin tone, the above syndromes all caused by insufficient liver blood	Quiet the spirit
	Fufang Zhenzhu Jiedu oral liquids	Traditional Chinese medicine	Nacre powder, roots of *Rehjnannia glutinosa*, rhizomes of *Smilax glabra*, bud or fresh flowers of *Lonicera japonica*, Carapace on the back and abdomen of *Chinemys reevesii*, roots and rhizomes of *Glycyrrhiza uralensis* or *Glycyrrhiza inflata* or *Glycyrrhiza glabra*	——	Light acne caused by heat toxins stasis blocking the skin; it can be seen rash; symptoms of the rash are mainly red papules, blackheads, or whiteheads, accompanied by a small amount of pus	Moisturize the skin and remove speckle
	Renshen Zhenzhu oral liquids	Traditional Chinese medicine	Roots and rhizomes of *Panax ginsen*g, pearl	2.5 g	Palpitations and insomnia, dizziness, forgetfulness, fatigue	Quiet the spirit
	Zhenqi Buxue oral liquids	Traditional Chinese medicine	Sheep placenta, pearl, roots and rhizomes of *Panax ginseng*, fruits of *Lycium barbarum*, roots of *Astragalus membranaceus* or *Astragalus membranaceus*, roots of *Angelica dahurica*, seed kernel of *Coix lacryma-jobi*, rhizomes of *Zingiber officinale*, fruits of *Ziziphus jujuba*. Excipients are protein sugar, sodium benzoate	12.5 g	Dizziness, pallor, fatigue, lack of qi and no desire to speak caused by deficiency of Qi and blood	Tranquilize
	Zhenzhu Qishe oral liquids	Traditional Chinese medicine	Pearl, *Agkistrodon acutus*, roots and rhizomes of *Panax ginsen*g, roots of *Polygonum multiflorum*, rhizomes of *Polygonatum kingianum* or *Polygonatum sibiricum* or *Polygonatum cyrtonema*	——	Deficiency of Qi and Yin, insufficiency of the liver and kidney, the symptoms can be seen as fatigue, lack of qi and no desire to speak, weakness of waist and knees, insomnia and dreaminess, dysphoria with feverish sensation in chest and palms as well as soles, spontaneous perspiration, night sweat, *etc*.	Quiet the spirit
**Ointments**	Bingshi Yushang ointments	Traditional Chinese medicine	Root and rhizomes of *Rheum palmatum* or *Rheum tanguticum* or *Rheum officinale*, Calamine, artificial gallstones of *Bos taurus domesticus*, pearl, Alum, gypsum fibrosum, artificial secretions in male sachets of *Moschus berezovskii* or *Moschus sifanicus* or *Moschus moschiferus*, crystallization of extracts of *Blumea balsamifera* or *Cinnamomum camphora*	——	Burning scalds, the area of burning scalds with degree II is less than 16%, the symptoms can be seen: local pain, blisters, edema. After removing the *epidermis*, the wound surface is moist or slightly damp, the wound base is bright red or pale, *etc*.	Detoxify and promote granulation
	Mayinglong Babao ointments	Traditional Chinese medicine	Calamine, amber, artificial secretions in male sachets of *Moschus berezovskii* or *Moschus sifanicus* or *Moschus moschiferus*, artificial gallstones of *Bos taurus domesticus*, pearl, crystallization of extracts of *Blumea balsamifera* or *Cinnamomum camphora*, Borax, Sal ammoniac	0.38 g	Red, swollen, and itchy eyes, lacrimation, red and erosive eyelid caused by disturbance of wind evil and fire evil upward; trachoma with the above-mentioned syndromes	Anti-inflammatory
	Zhenggu ointments	Traditional Chinese medicine	Roots of *Angelica sinensis*, seeds of *Momordica cochinchinensis*, roots of *Codonopsis pilosula* or *Codonopsis pilosula* or *Codonopsis tangshen*, roots of *Arnebia euchroma* or *Arnebia guttata*, roots of *Saposhnikovia divaricata*, tubers of *Corydalis yanhusuo*, peel of *Citrus reticulata*, roots of *Rehjnannia glutinosa*, roots and rhizomes of *Notopterygium incisum* or *Notopterygium franchetii*, fruits of *Lycium barbarum*, skin of *Elephas maximus*, roots of *Paeonia lactiflora* or *Paeonia veitchii*, roots of *Cyathula officinalis*, rhizomes of *Atractylodes lancea* or *Atractylodes chinensis*, roots and rhizomes of *Panax ginsen*g, bone of *Panthera pardus* or *Neofelis nebulosa* or *Panthera uncia*, bark of *Aralia chinensis*, processed products of daughter roots of *Aconitum carmichaelii*, tubers of *Gastrodia elata*, rhizomes of *Polygonatum odoratum*, *Bombyx mori* that infected *Beauveria bassiana*, roots of *Angelica dahurica*, fruits of *Psoralea corylifolia*, rhizomes of *Atractylodes macrocephala*, roots of *Astragalus membranaceus* or *Astragalus membranaceus*, whole plant of *Speranskia tuberculata*, fleshy stems of *Cynomorium songaricum*, roots and rhizomes of *Rheum palmatum* or *Rheum tanguticum* or *Rheum officinale*, roots of *Polygala tenuifolia* or *Polygala sibirica*, leaves of *Epimedium brevicomu* or *Epimedium sagittatum* or *Epimedium pubescens* or *Epimedium koreanum*, roots of *Angelica pubescens*, roots of *Paeonia lactiflora*, roots of *Dipsacus asper*, tubers of *Pinellia ternata*, rhizomes of *Dioscorea hypoglauca*, roots and tubers of *Asarum heterotropoides* or *Asarum sieboldii* or *Asarum sieboldii*, roots of *Gentiana macrophylla* or *Gentiana straminea* or *Gentiana crassicaulis* or *Gentiana dahurica*, roots of *Aconitum carmichaelii*, rhizomes of *Zingiber officinale*, tubers of *Arisaema erubescens* or *Arisaema heterophiles* or *Arisaema amurense*, rhizomes of *Dioscorea opposita*, roots of *Aconitum kusnezoffii*, roots bark of *Paeonia suffruticosa*, rhizomes of *Alisma orientate* or *Alisma plantago-aquatica*, fruits of *Chaenomeles speciosa*, flowers of *Carthamus tinctorius*, heartwood of *Dalbergia odorifera*, root bark of *Acanthopanax gracilistylus*, roots of *Morinda officinalis*, roots of *Rehjnannia glutinosa*, heartwood of *Caesalpinia sappan*, Crinis carbonisatus (carbonized human hair), rhizomes of *Drynaria fortunei*, bark of *Cinnamomum cassia*, roots and Rhizomes of *Panax notoginseng*, fleshy stems with scale leaves of *Cistanche deserticola* or *Cistanche tubulosa*, resin of *Commiphora myrrha* or *Commiphora molmol*, fruits of *Citrus medica*, tubers of *Bletilla striata*, gallstones of *Bos taurus domesticus*, resin of *Boswellia carterii* or *Boswellia bhaw-dajiana*, *Manis pentadactyla*, *Drgonsbones* (fossils of mammalian bones), resin of fruits of *Daemonorops draco*, roots bark of *Lycium chinense* or *Lycium barbarum*, secretions in male sachets of *Moschus berezovskii* or *Moschus sifanicus* or *Moschus moschiferus*, soft extract without bark, branches, and trunk of *Acacia catechu*, young antlers of male deer of *Cervus nippon* or *Cervus elaphus*, Crystallization of extracts of *Blumea balsamifera* or *Cinnamomum camphora*, pearl, roots of *Aucklandia lappa*	25 g	Arthralgia and myalgia, injuries from falls	Promote granulation and promote bone growth and regeneration
	Longzhu ointments	Traditional Chinese medicine	Artificial secretions in male sachets of *Moschus berezovskii* or *Moschus sifanicus* or *Moschus moschiferus*, borax, calamine (calcined), Sal ammoniac, crystallization of extracts of *Blumea balsamifera* or *Cinnamomum camphora*, artificial gallstones of *Bos taurus domesticus*, pearl (concocted), amber	——	Furunculosis, redness, swelling, heat, pain, and mild scalding	Anti-inflammatory
	Yangxin Dawayimi Xikemi ointments	Uyghur medicine	Secretions in male sachets of *Moschus berezovskii* or *Moschus sifanicus* or *Moschus moschiferus*, heartwood of *Santalum album*, pearl, resin of *Pistacia lentiscus*, bark of *Cinnamomum cassia*, flowers of *Rumex dentatus*, silkworm cocoons, wood of *Aquilaria sinensis*, stigma of *Crocus sativus*, whole plant or root bark of *Operculina turpethum* or *Convolvulus turpethum* or *Ipomoea turpethum*, dried mass of exudate of *Bambusa textilis* or *Schizostachyum chinense*, fruits of *Berberis kansuensis* or *Berberis dasystachya*, roots of *Limonium gmelinii*	8 g	Cardiothoracic pain, palpitations, stomach deficiency, visual weakness, and neurasthenia	Quiet the spirit
**Granules**	Fufang Zhenzhu Kouchuang granules	Traditional Chinese medicine	Pearl, fruits of *Schisandra chinensis*, rhizomes of *Atractylodes lancea* or *Atractylodes chinensis*, roots and rhizomes of *Glycyrrhiza uralensis* or *Glycyrrhiza inflata* or *Glycyrrhiza glabra*	——	Heart and spleen damp heat type aphthous (recurrent oral ulcer); the symptoms can be seen as aphthous: redness and swelling around, sunken in the middle, yellow and white surface , burning pain, dry mouth and bad breath, red tongue	Promote granulation and anti-ulcer
	Jingxin granules	Traditional Chinese medicine	Root and rhizomes of *Salvia miltiorrhiza*, roots of *Paeonia lactiflora*, roots of *Rehjnannia glutinosa* (concocted), fruits of *Gardenia jasminoides*, pearl powder, roots of *Angelica sinensis*	——	Hot sweating induced by Yin deficiency liver thriving in menopausal women, dizziness and tinnitus, irritability, weakness of waist and knees, insomnia, and dreaminess	Quiet the spirit
	Nvzhen granules	Traditional Chinese medicine	Fruits of *Ligustrum lucidum*, overground part of *Eclipta prostrata*, roots of *Rehjnannia glutinosa*, roots of *Arnebia euchroma* or *Arnebia guttata*, seeds of *Ziziphus jujuba*, seed kernel of *Platycladus oriental*, stems and branches of *Uncaria rhynchophylla* or *Uncaria macrophylla* or *Uncaria hirsuta* or *Uncaria sinensis* or *Uncaria sessilifructus*, pearl powder, sclerotium of *Poria cocos*, young leaves and radicles in seeds of *Nelumbo nucifera*	16.7 g	Menopause syndrome accompanied by liver kidney Yin deficiency, heart and liver fire thriving; the symptoms can be seen as hot sweating, dysphoria with feverish sensation in chest and palms as well as soles, palpitations, insomnia	Quiet the spirit
	Xinxue granules	Traditional Chinese medicine	Magnetite, gypsum fibrosum, talcum, calcite, niter, mirabilite, fruits of *Gardenia jasminoides*, leaves of *Phyllostachys glauca*, roots of *Serratula chinensis*, overground part of *Andrographis paniculata*, nacre powder, wood of *Aquilaria sinensis*, artificial gallstones of *Bos taurus domesticus*, crystallization of extracts of *Blumea balsamifera* or *Cinnamomum camphora*	54 g	Exogenous pyreticosis, heat evil thriving, and excessive syndrome; it can be seen as high fever, irritability, tonsillitis, upper respiratory tract infection, tracheitis, cold accompanied by the above-mentioned syndromes	Detoxifing and anti-inflammatory
	Zhenhong granules	Traditional Chinese medicine	Pearl, roots, and rhizomes of *Panax ginseng*, Root of *Rehjnannia glutinosa*, Sporophore of *Ganoderma lucidum* or *Ganoderma sinense*, rhizomes of *Polygonatum odoratum*, roots of *Polygonum multiflorum*, roots and rhizomes of *Salvia miltiorrhiza*, overground part of *Leonurus japonicus*	——	Lusterless complexion, dark and gloomy spots caused by deficiency of Qi and blood, insufficient kidney Yin, Qi stagnation, and blood stasis	Remove speckle
**Eye drops**	Bingzhen Qingmu eye drops	Traditional Chinese medicine	Nacre powder, sinc gluconate, crystallization of extracts of *Blumea balsamifera* or *Cinnamomum camphora*	——	Pseudomyopia and asthenopia in adolescents	Improve eyesight
	Jinzhen eye drops	Traditional Chinese medicine	Bud or fresh flowers of *Lonicera japonica*, bud and inflorescence of *Buddleja officinalis*, capitulum of *Chrysanthemum indicum*, overground part of *Mentha haplocalyx*, pearl, crystallization of extracts of *Blumea balsamifera* or *Cinnamomum camphora*	——	Chronic catarrhal conjunctivitis belongs to wind heat stagnated in eyes syndrome; the symptoms can be seen as redness in the eyelids, light and tears, eye burning and itching pain, dry eyes, asthenopia, *etc*.	Improve eyesight and anti-inflammatory
	Shezhu Mingmu eye drops	Traditional Chinese medicine	Pearl, secretions in male sachets of *Moschus berezovskii* or *Moschus sifanicus* or *Moschus moschiferus*, *Cordyceps sinensis*, shells of *Haliotis diversicolo*r or *Haliotis discus hannai* or *Haliotis ovina* or *Haliotis ruber* or *Haliotis asinina* or *Haliotis laevigata* (calcined), rhizomes of *Coptis chinensis* or *Coptis deltoidea* or *Coptis teeta*, bark of *Phellodendron chinense*, roots and rhizomes of *Rheum palmatum* or *Rheum tanguticum* or *Rheum officinale*, crystallization of extracts of *Blumea balsamifera* or *Cinnamomum camphora*, bile of *Zaocys dhumnades*, porcine bile (gallbladder) ointment, calamine (calcined), leaves or twig of *Perilla frutescens*, overground part of *Schizonepeta tenuifolia*	——	Senile cataracts in the primary and middle stages, asthenopia; the symptoms can be seen as ocular tiredness, eye swelling and pain, dry eyes, blurred vision	Improve eyesight and remove nebula
	Siwei Zhenceng Bingpeng eye drops	Traditional Chinese medicine	Nacre powder, natural crystallization of extracts of *Blumea balsamifera* or *Cinnamomum camphora*, Borax, boric acid	——	Distal vision declines in adolescents, cannot see too long, caused by insufficient liver Yin and liver Qi abnormally exuberant; pseudomyopia and asthenopia in adolescents	Improve eyesight
	Zhenzhu Mingmu eye drops	Traditional Chinese medicine	Pearl liquid, crystallization of extracts of *Blumea balsamifera* or *Cinnamomum camphora*	20 ml	Asthenopia and chronic conjunctivitis	Improve eyesight and remove nebula
**Suppositories**	Angong Niuhuang suppositories	Traditional Chinese medicine	Gallstones of *Bos taurus domesticus*, horn of *Bubalus bubalis*, Secretions in male sachets of *Moschus berezovskii* or *Moschus sifanicus* or *Moschus moschiferus*, pearl, cinnabar, realgar, rhizomes of *Coptis chinensis* or *Coptis deltoidea* or *Coptis teeta*, roots of *Scutellaria baicalensis*, fruits of *Gardenia jasminoides*, roots of *Curcuma wenyujin* or *Curcuma longa* or *Curcuma kwangsiensis* or *Curcuma phaeocaulis*, crystallization of extracts of *Blumea balsamifera* or *Cinnamomum camphora*	15 g	Pyreticosis, evil into the pericardium, febrile convulsion, delirium, stroke coma, encephalitis, meningitis, toxic encephalopathy	Quiet the spirit
	Fuyanping suppositories	Traditional Chinese medicine	Roots of *Sophora flavescens*, fruits of *Cnidium monnieri*, branches and leaves of *Picrasma quassioides*, nacre powder, crystallization of extracts of *Blumea balsamifera* or *Cinnamomum camphora*, Alum (calcined), extracts of stems and leaves of *Mentha haplocalyx* (l-Menthol), boric acid, berberine hydrochloride	2.5 g	Damp heat pouring downward, belt channel dysfunction, abnormal leucorrhea, itching and swelling of the vulva, vaginitis, and vulvitis caused by trichomonad, mold, and bacteria	Anti-inflammatory
	Shexiang Zhichuang suppositories	Traditional Chinese medicine	Artificial secretions in male sachets of *Moschus berezovskii* or *Moschus sifanicus* or *Moschus moschiferus*, pearl, crystallization of extracts of *Blumea balsamifera* or *Cinnamomum camphora*, Calamine powder, roots and rhizomes of *Panax notoginseng*, fruits of *Schisandra chinensis*, artificial gallstones of *Bos taurus domesticus*, extract made from whole plant of *Atropa belladonna*	——	Stool bleeding, bloody redness and burning pain in the anus caused by thriving heat in the large intestine; various types of hemorrhoids and anal fissures accompanied by the above-mentioned syndromes	Anti-inflammatory
	Xiaozhi suppositories	Traditional Chinese medicine	*Drgonsbones* (fossils of mammalian bones) (calcined), calomel, crystallization of extracts of *Blumea balsamifera* or *Cinnamomum camphora*, pearl (concocted)	41 g	Internal and external hemorrhoids	Anti-inflammatory
**Drop pills**	Lianqin Zhenzhu drop pills	Traditional Chinese medicine	Fruits of *Forsythia suspensa*, roots of *Scutellaria baicalensis*, fruits of *Gardenia jasminoides*, powder, clumps or granules made from leaves or stems and leaves of *Baphicacanthus cusia* or *Polygonum tinctorium* or *Isatis indigotica*, gypsum fibrosum (calcined), nacre powder, artificial gallstones of *Bos taurus domesticus*, roots and rhizomes of *Glycyrrhiza uralensis* or *Glycyrrhiza inflata* or *Glycyrrhiza glabra*, extracts of stems and leaves of *Mentha haplocalyx* (l-menthol), crystallization of extracts of *Blumea balsamifera* or *Cinnamomum camphora*	——	Recurrent aphthous ulcer (mild aphthous ulcer or stomatitis aphthous) caused by syndrome of heart and spleen accumulated heat; the symptoms can be seen as oral ulcer and pain accompanied by dysphoria, hot and dry mouth, red and dry tongue, the coating on the tongue is yellow and greasy, fine rapid stringlike pulse, *etc*.	Anti-ulcer
**Effervescent tablets**	Fuyanping Yindao effervescent tablets	Traditional Chinese medicine	Roots of *Sophora flavescens*, nacre powder, berberine hydrochloride, branches and leaves of *Picrasma quassioides*, crystallization of extracts of *Blumea balsamifera* or *Cinnamomum camphora*, boric acid, fruits of *Cnidium monnieri*, extracts of stems and leaves of *Mentha haplocalyx* (l-menthol), Alum (calcined)	2.5 g	Bacterial, mycotic, trichomonal vaginitis, vulvar itching, cervicitis, pelvic inflammatory disease, abnormal leucorrhea, urinary tract infection	Anti-inflammatory
**Lozenges**	Babao Wudan Yaomo	Traditional Chinese medicine	Horn of *Bubalus bubalis*’s concentrated powder, horn of *Saiga tatarica*, secretions in male sachets of *Moschus berezovskii* or *Moschus sifanicus* or *Moschus moschiferus*, crystallization of extracts of *Blumea balsamifera* or *Cinnamomum camphora*, pearl, secretions of *Bufo bufo gargarizans* or *Bufo melanostictus*, gallstones of *Bos taurus domesticus*, cinnabar, gallbladder, or bile of *Bos taurus domesticus* or *Bubalus bubalis*, gallbladder of *Ursus thibetanus* or *Ursus arctos*, gallbladder of *Zaocys dhumnades*, gallbladder of *Sus scrofa domestica*, rhizomes of *Ligusticum chuanxiong*, gallbladder of *Mylopharyngodon piceus*, rhizomes of *Nelumbo nucifera*, flowers of *Carthamus tinctorius*, overground part of *Cirsium setosum*, overground part of *Cirsium japonicum*, rhizomes of *Imperata cylindrica*, fruit clusters of *Prunella vulgaris*, root bark of *Paeonia suffruticosa*, bud of *Eugenia caryophyllata*	120 g	Vomiting blood, coughing up blood, epistaxis, hematochezia, dysentery with red and white feces, carbuncle sores, innominate swelling ulcer (it is a poisoned sore that is neither like gangrene, nor carbuncle, nor malignant boils), stubborn ringworm, dermatitis, eczema, *etc*.	Anti-inflammatory, moisturize the skin, and remove speckle
**Mixtures**	Xiaoer Jianpikaiwei mixtures	Traditional Chinese medicine	Roots of *Astragalus membranaceus* or *Astragalus membranaceus*, rhizomes of *Atractylodes macrocephala*, rhizomes of *Dioscorea opposita*, fruits of *Ziziphus jujuba*, fruits of *Crataegus pinnatifida* or *Crataegus pinnatifida*, seeds of *Nelumbo nucifera*, fruits of *Lycium barbarum*, peel of *Citrus reticulata*, nacre powder, roots and rhizomes of *Glycyrrhiza uralensis* or *Glycyrrhiza inflata* or *Glycyrrhiza glabra*, honey	——	Infantile anorexia and indigestion caused by weakness of spleen and stomach, and promoting calcium absorption in children	An auxiliary role in digestion
**Aerosols**	Yansukang aerosols	Traditional Chinese medicine	Artificial gallstones of *Bos taurus domesticus*, pearl, realgar, secretions of *Bufo bufo gargarizans* or *Bufo melanostictus*, secretions in male sachets of *Moschus berezovskii* or *Moschus sifanicus* or *Moschus moschiferus*, crystallization of extracts of *Blumea balsamifera* or *Cinnamomum camphora*	——	Lung-stomach excess heat syndrome accompanied by sore throat and tonsillitis	Anti-inflammatory
**Oils**	Zibing oils	Traditional Chinese medicine	Roots of *Arnebia euchroma* or *Arnebia guttata*, stems and branches of *Lonicera japonica*, crystallization of extracts of *Blumea balsamifera* or *Cinnamomum camphora*, pearl, roots of *Angelica sinensis*	——	Superficial skin ulcer (belongs to Yang syndrome)	Anti-ulcer, moisturize the skin

“——” means that the dose of pearls has not been found or disclosed.

According to our statistics and analysis, the indications of preparations containing pearls involve diseases of 17 systems (see Table 4 for details). The common disease types and frequencies in order are symptoms or physical signs and clinical or abnormal laboratory findings that cannot be classified elsewhere (29.27%), digestive system diseases (14.15%), respiratory system diseases (11.85%), nervous system diseases (8.58%), skin and subcutaneous tissue diseases (7.07%), eye and adnexa diseases (6.45%), circulatory system diseases (5.57%), diseases of the genitourinary system (3.98%), musculoskeletal system and connective tissue diseases (3.89%), and characteristic diseases of traditional Chinese medicine (ethnic medicine) (2.65%), among others. Because of the remarkable effect of pearls, these preparations have great advantages in the treatment of certain diseases, such as skin (Xiao et al., 2015) and eye (Niu and Xu, 2005) diseases.

**TABLE 4:** Classification statistics table for the indications of preparations containing pearls.

Diseases Classification	Name of diseases (the number in brackets is the number of Preparations used to treat the disease)	Number of times used	Percentage (%)
**Symptoms or physical signs and clinical or laboratory abnormal findings that cannot be classified elsewhere**	Headache vertigo (20), shortness of breath (14), fatigue (14), palpitations (13), chest tightness (11), dysphoria (11), febrile convulsions (10), hemiplegia (9), palpitations and insomnia (9), headache (8), fever (8), high fever (8), loss of appetite (7), insomnia (7), dizziness (7), pediatric convulsions (9), insomnia and dreaminess (6), convulsions (13), chest pain (7), pain (4), hand and foot twitching (4), common cold with fever (3), unconscious (4), dysphoria with smothery sensation (3), insomnia amnesia (3), the complexion is bleak (3), dizziness and insomnia (3), dysphoria (3), impatient and irritable (3), red tongue and yellow coating (3), pediatric internal thermal (3), liver depression and Qi stagnation (2), spontaneous sweating (2), headache insomnia (2), high fever does not go away (2), conscious coma (2), jaundice (2), tired (2), light headedness (2), belt channel dysfunction (2), hemoptysis (2), speech mania (2), dry tongue and vertigo (2), dysphasia (2), amnesia (2), infantile acute febrile seizures (2), irritability to thirst (2), lack of Qi and no desire to speak (2), hot and sweaty (2), crazy slapstick (2), night cry and startled (2), dysphoria with feverish sensation in chest and palms as well as soles (2), confusing and ravings (1), thoracic diaphragmatic stagnant cold (1), blood stasis (1), mania (1), low back pain and fatigue (1), cold hands and feet (1), edema (1), distending pain in the thoracic flank (1), incoherent speech (1), the impermanence of crying and laughing (1), redness, swelling and pain (1), deficiency of both Qi and blood (1), general fatigue (1), chills and cold limbs (1), asthenia and spontaneous sweating (1), children with phlegm-heat internal closure (1), the body and face are hot (1), acute convulsion caused by wind heat (1), restless sleep at night (1), spirits droop (1), yellow and greasy tongue coating (1), Yang deficiency of the spleen and kidney (1), heart and liver heat thriving (1), deficiency of essential Qi (1), bitter mouth and yellow urine (1), feeling of fullness in the head (1), deficiency of Qi and blood (1), hot flashes after the afternoon (1), night sweats (1), high fever and polydipsia (1), thirsty (1), delirium goes crazy (1), vigorous liver fire (1), neurotic mania (1), liver Yang induces vertigo (1), dysphoria and insomnia (1), arthralgia aggravated by cold (1), seasickness (1), carsickness (1), restlessness (1), acute convulsions in children caused by high fever (1), pediatric internal heat caused by fire evil (1), cold and fever (1), liver stomach discord (1), headache caused by brain heat (1), headache caused by blood heat (1), heat accumulation in the viscera of children (1), face and lips are red (1), not tranquil caused by body hot (1), fainting due to heatstroke (1), depression with heat syndrome (1), unhealthy skin tone (1), pale (1), deficiency of Qi and Yin (1), insufficient liver and kidney (1), spontaneous sweats and night sweats (1), mental burnout (1), hematoma (1), vexation and irritable (1), hematemesis (1)	331	29.35
**Digestive system diseases**	Gastric and duodenal ulcer (8), vomiting hiccups (7), swollen and painful gums (7), constipation (6), sores mouth and tongue (5), gastral cavity distending pain or tingling (5), stomachache (5), peptic ulcer (5), aphthous (5), belching and acid regurgitation (4), abdominal distension and loose stools (4), abdominal distension and pain (4), esophageal cancer (4), dysphagia (3), stomach cancer (3), oral ulcer (3), hemorrhoids (3), acute and chronic hepatitis (2), tongue swelling and pain (2), esophageal stricture and obstruction (2), dysphagia and salivated (2), vomiting and diarrhea (2), indigestion (2), bitter and dry mouth (2), nausea (2), chronic gastritis (2), chronic hepatitis (2), hematochezia (2), recurrent oral ulcer (2), gangrenous stomatitis (2), anal fissure (2), stool bleeding (2), trismus (2), liver pain (1), cirrhosis (1), hepatotoxicity (1), liver seepage (1), cholecystitis (1), bitter mouth and dry throat (1), gastroenteritis (1), acute abdominal pain (1), esophagitis (1), anorexia (1), costal pain and abdominal distension (1), lower abdomen distending pain (1), acute cholecystitis (1), heart and liver heat thriving (1), uncomfortable lateral thorax (1), pain in the gastric cavity (1), erosive gastritis (1), difficulty in defecation (1), angular salivation (1), hematemesis (1), redness and swelling of the uvula (1), oral erosion (1), pantothenic acid (1), acid regurgitation (1), vomiting and salivation (1), colon ulcer (1), abdominal pain (1), diarrhea with abdominal pain (1), toothache (1), acute gastroenteritis (1), mouth sore swelling and pain (1), eruptive stomatitis (1), burning and dry mouth (1), gingival decay (1), mouth and tongue swelling and pain (1), erosions of the tongue and mouth (1), gingivitis (1), ulcerated gingiva (1), bleeding between teeth (1), abdominal pain and vomiting (1), stomach deficiency (1), dry mouth and bad breath (1), red tongue (1), burning pain in the anus (1), recurrent aphthous (1), hot and dry mouth (1), infantile anorexia (1)	160	14.15
**Respiratory system diseases**	Sore throat (35), tonsillitis (12), acute and chronic pharyngitis (7), cough (4), upper respiratory tract inflammation (4), tracheitis (4), chronic bronchitis (4), pharyngitis (3), pharyngolaryngitis (3), colds at the begin stage (3), bronchial asthma (3), pharyngitis (3), cough with excessive phlegm (3), shortness of breath (3), rotten throat (2), dry and burning throat (2), acute tonsillitis (2), redness and swelling of the larynx nucleus (1), external evil induces wind cold (1), pant and cough of prolonged illness (1), coughing weakness (1), less phlegm but sticky (1), blood in the sputum (1), lung cancer (1), infantile pant and cough caused by lung heat (1), spitting yellow and thick (1), chronic senile bronchitis (1), chronic pharyngitis (1), chronic laryngitis (1), dry throat (1), laryngeal itching (1), aphonia (1), effortful vocalization (1), dysphonia (1), redness and swelling of the lateral pharyngeal cord (1), acute and chronic rhinitis (1), sinusitis (1), nasal congestion (1), rhinorrhea (1), coughing with nasal congestion (1), red eyes, swelling and pain (1), rhinitis (1), throat erosion (1), body heat and cough (1), infantile tracheitis (1), bronchitis (1), asthmatic bronchitis (1), cough and phlegm (1), foreign body sensation in the throat (1), pharyngitis and oral mucosal ulcer (1), red and swollen tonsils (1), acute pharyngitis (1), upper respiratory tract infection (1), epistaxis (1)	134	11.85
**Nervous system diseases**	Delirium (13), stroke coma (12), skewing of the mouth and eyes (6),encephalitis (5), meningitis (5), toxic encephalopathy (5), epilepsy (4), paralysis (4), stroke hemiplegia (4), neuropathic pain/neuropathic headache (4), numbness in limbs (3), cerebral hemorrhage (2), epilepsy (2), slurred speech (2), nerve palsy (2), psychoneurosis (2), epileptic mania (2), brain pain (2), body numbness (1), concussion (1), neuropathic disorders (1), neck stiffness and brain distension (1), cerebral thrombosis (1), polio (1), various neuritis (1), dementia with epilepsy (1), mental decline (1), cerebral thrombosis recovery period and sequelae (1), cerebral arteriosclerosis (1), convulsive epilepsy (1), stroke with phlegm (1), memory loss (1)	93	8.58
**Skin and subcutaneous tissue diseases**	Acne (8), carbuncle and boils swelling (5), skin eczema (4), chloasma (4), innominate swelling ulcer (it is a poisoned sore that is neither like gangrene, nor carbuncle, nor malignant boils) (4), boils (3), the first onset of carbuncle and boils swelling (3), dermatitis (3), vitiligo (3), early white hair (2), furuncle carbuncle sores (2), ulcer (2), carbuncle gangrene sores boils (2), sores and hot boils (2), pterygium (2), skin dryness (2), spots and ulcers (1), the sores not close for a long time (1), the sores at the begin stage (1), furuncle on the back (1), skin gaunt (1), pharyngitis with furuncle (1), carbuncle swelling (1), infantile hot furunculosis (1), carbuncle ulcer furuncle (1), furuncle pain (1), suppuration (1), the skin color is chlorosis (1), contact dermatitis (1), sore pain and swelling at the beginning (1), scabies ulceration (1), maculopathy (1), mold infection (1), carbuncle (1), sore ulceration (1), skin rash (1), red papules (1), perianal eczema (1), freckles (1), allergic tingling and itching (1), dark and gloomy spots (1), ulceration, swelling and pain (1), furuncle (1), redness, swelling, heat and pain (1), superficial skin ulcer (1)	80	7.07
**Eye and adnexa diseases**	Cataract (6), eyelid red erosions (5), pinkeye (4), asthenopia (4), trachoma (3), epiphora with wind (3), corneal opacity (3), dry eyes (3), cloudy nebula (3), red, swollen, painful, and itchy eyes (3), dizziness (2), keratitis (2), trachoma (2), lacrimation (2), pseudomyopia (2), blurred vision (2), vision fatigue (2), red eyes (1), senile vision decline (1), spots nebula (1), conjunctivitis (1), vitreous opacity (1), retinopathy (1), senile lacrimation (1), senile ocular hypofunction (1), red, swollen, and rotten eyes (1), afraid of seeing the light (1), visually weak (1), eyes edge prickling and itching (1), chronic catarrhal conjunctivitis (1), redness in the eyelids (1), see the light and shed tears (1), black of eye with cloudy nebula (1), eyes burning, itching, and pain (1), eyes swelling and pain (1), cannot look at for a long time (1), decreased far visual acuity (1), chronic conjunctivitis (1), ocular hypofunction in old age (1)	73	6.45
**Circulatory system diseases**	Hypertension (20), angina (13), coronary heart disease (10), heartache (8), irregular blood pressure (2), cardiac dysfunction (2), heart disease (2), arrhythmia (2), myocardial infarction (1), tachycardia or bradycardia (1), phlegm obstructed to the heart (1), cardiothoracic pain (1)	63	5.57
**Diseases of the genitourinary system**	Vaginitis (4), abnormal leucorrhea (4), vulvitis (3), mammary carbuncle swelling and pain (2), urinary tract infection (2), frequent nocturia (2), damp heat pouring downward (2), itching and swelling of the vagina (2), cervicitis (2), breast cancer (2), menorrhagia (2), irregular menstruation (1), mammary carbuncle (1), excessive leucorrhea (1), hematuria (1), cold and heat kidney disease (1), insufficient kidney essence (1), kidney damage and pulse injury (1), kidney disease (1), yellow and red urine (1), urethral burning and stinging pain (1), acute cholecystitis (1), increased nocturia (1), uterine bleeding (1), short red urine (1), menstrual color dark red (1), prolonged menstrual periods (1), itching of the vulva (1), pelvic inflammatory disease (1)	45	3.98
**Musculoskeletal system and connective tissue diseases**	Sore waist and weak legs/sore waist and knees (8), sore muscles and bones (3), joints pain (2), rheumatism (2), rheumatoid (2), osteonecrosis (1), vasculitis (1), tenosynovitis (1), unfavorable limb and joints (1), nape rigidity (1), weak waist and legs (1), arthralgia and myalgia (1), muscles atrophy (1), red, swollen, hot, and painful joints (1), pulse disease (1), pain caused by wind, cold, and dampness (1), hand and foot spasm (1), neck discomfort (1), unsteady walking (1), rib pain (1), muscles pain (1), stiff neck with twitching (1), joints and muscles swelling and pain (1), acute soft tissue and other chronic tissue damage (1), lumbar muscles strain (1), joints contusion (1), frozen shoulder (1), cervical spondylosis (1), lumbar disc herniation (1), red tendons (1), local tissue swelling (1), chapped hands and feet (1)	44	3.89
**Characteristic diseases of traditional Chinese medicine (ethnic medicine)**	Phlegm and saliva are thriving (11), *Danshuang Rue* (unilateral or bilateral tonsillitis) (7), evil into the pericardium (5), *Baimai* diseases (3), *Longxue* disorder (1), infantile phlegm thriving caused by wind (1), phlegm thriving caused by fire (1), old nebula (after the black of eye disease is healed, it forms a scar, with a smooth surface, clear edges, and no red pain as the main manifestation, which is a pannus disease) (1), *Baizhang* (cataract) (1), *Rue* (tonsillitis) (1), hot evil thriving (1), *Heibaimai* diseases (1)	34	2.65
**Injury, poisoning, and certain other consequences of external causes**	Pyreticosis (9), burning scalds (5), poisoning (2), injuries from falls (2), consumptive disease with injury (1), swelling and pain after insect bite (1), wind heat (1), residual sores surface (1)	22	1.86
**Certain infectious diseases and parasitic diseases**	*Dansha* (an acute respiratory infectious disease) (3), leprosy (2), diphtheria (2), epidemic infection (1), typhoid fever (1), miasmatic infection (1), syphilis (1), anthrax (1), lague fever (1), infectious viral hepatitis (1), *tuberculosis* (1), mumps (1), tinea corporis (1), athlete’s foot (1), chancre rot (1)	19	1.68
**Mental and behavioral disorders**	Neurasthenia (6), delirium (4), panic and dysphoria (1), fear of uneasiness (1), insanity (1)	13	1.15
**Ear and mastoid diseases**	Tinnitus (9), ear distension and tinnitus (1), Meniere’s disease (1)	11	0.97
**Endocrine, nutritional, and metabolic diseases**	Hyperlipidemia (5), hypothyroidism (1), diabetes (1)	7	0.62
**Tumor**	Tumor (1)	1	0.09
**Diseases of blood and hematopoietic organs and certain diseases involving immune mechanisms**	Anemia (1)	1	0.09
**Total**		1,131	100

Note: the percentage is calculated based on the total number of times “1,131” is used.

## 3 Chemical Constituents of Pearls

**FIGURE 1:**
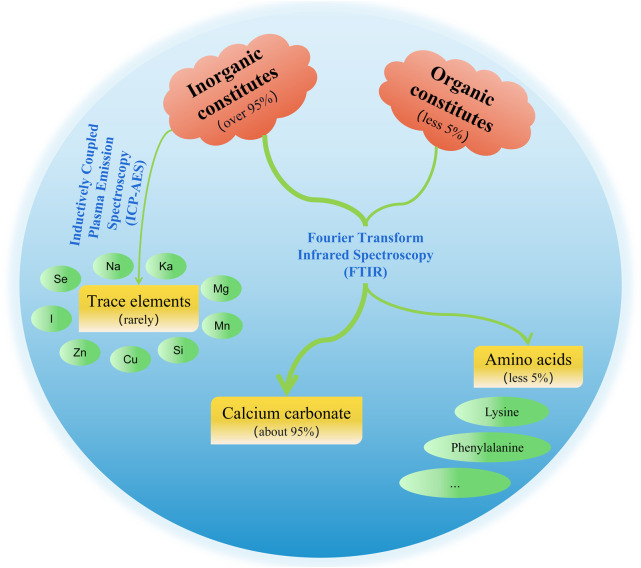
Schematic diagrams of chemical constituents and test methods of pearls.

The chemical constituents of pearls include inorganic constituents, organic constituents, and water (see Figure 1 for details) (Ouyang et al., 2012). Inorganic constituents, mainly calcium carbonate, account for more than 95% of pearls content, and there are a variety of trace elements; the content of organic constituents is low, mainly composed of proteins and polysaccharide-like substances; and the water content is less than 2% (Wu, 2017).

### 3.1 Inorganic Constituents of Pearls

#### 3.1.1 Calcium Carbonate

Calcium-like substances are abundant in pearls, and calcium carbonate, in turn, is the main component in calcium type of pearls, accounting for about 95% of the whole calcium-like substances (Yang et al., 2004). The crystalline phase of calcium carbonate is mainly aragonite, with a small amount of calcite and vaterite. Aragonite and calcite are common in nature, whereas vaterite is the most unstable state of calcium carbonate and rare in nature. The nacre is mainly composed of an aragonite layer but contains a small amount of calcite, namely, the prismatic layer (Ouyang et al., 2012). Calcium-like substances based on calcium carbonate can prevent and treat various diseases caused by calcium deficiency, such as rickets, osteoporosis, and dementia. They also have good osteoconductivity and osteogenic effects, which can be used as an alternative material to biological bone and are a good health care product (Zhan, 2010; Li et al., 2015).

#### 3.1.2 Trace Elements

The contents of trace elements in the human body are extremely small, but they have powerful biological effects. They are involved in the metabolic process of enzymes, hormones, vitamins, and nucleic acids (Yang, 2008). Pearls contain more than 10 kinds of trace elements such as Na, K, Mg, Mn, Si, Cu, Fe, Zn, Ba, Ge, Cr, Ni, Co, Ti, Sc, Se, Br, I, and Pb (Li, 2009; Xue and Xu, 2013), of which a large part are essential trace elements for the human body. Studies have found that the different types and content of trace elements can directly affect the treatment efficacy, the color, and the quality of pearls (Yang et al., 2004). However, the types and content of trace elements in pearls largely depend on their growth environment; for example, Na, K, Mg, and Sr are the main enriched elements in seawater pearls, whereas Mn and Ba are the main enriched elements in freshwater pearls (Li, 2009). Moreover, there is a certain regularity for the enriched elements in seawater and freshwater pearls; that is, the contents of Na, Mg, S, and Sr in seawater pearls are high, and the content of Mn is low, whereas freshwater pearls show the opposite trend (Zhang, 2019). This indicates the differences in trace elements in pearls under different environments. The colors of pearls are also related to their element types and content. For example, pearls with Mg and Mn^2+^ elements exhibit purple color, those with Fe^3+^ exhibit orange color, and those with organic constituents exhibit black color (Li, 2009). Freshwater pearls contain a higher content of Mn, indicating that the Mn element is ubiquitous in freshwater pearls. Studies have found no significant difference in the content of metallic elements with the same types in white and purple pearls, so they might not be the main factor contributing to the color of pearls. Paradoxically, this experiment also similarly shows that Fe and Mn are most abundant in purple pearls, whereas Fe is not detected in white pearls, leading to the conclusion that the darker the color, the higher the contents of Fe and Mn (Jiang et al., 2019). It shows that the relationship between trace elements and pearls’ color is controversial and needs to be deeply studied and explored.

The trace elements of pearls exert their effects on the human body mainly as follows: Se can enhance human immunity and has an anti-cancer effect; Zn can activate human superoxide dismutase (SOD), thereby clearing peroxidized lipids that predispose to human aging; Mn can protect against cardiovascular diseases and regulate the nervous system, promoting the absorption of Ca in the human body; Ge has an anti-tumor effect; Fe can be used to improve anemia symptoms (Dong et al., 2011; Li et al., 2015). Although trace elements play an indispensable role in the human body because most of them are metals or even heavy metal elements, their excessive levels can also harm the body. The 2020 edition of *The Pharmacopoeia of the People’s Republic of China* stipulates that the heavy metals and harmful elements in pearls shall not exceed 5 mg/kg for Pb, 0.3 mg/kg for Cd, 2 mg/kg for As, 0.2 mg/kg for Hg, and no more than 20 mg/kg for Cu (Committee, 2020). Although the contents of other metal elements in pearls are not prescribed, we should focus on their intake when they are used. Excessive intake can still cause harm to the human body. For example, excessive Fe intake can cause vomiting, diarrhea, melena, gastroenteritis, and even comatose. When the body ingests too much Fe, it will form goiter and induce hyperthyroidism and thyroid cancer; high intake of Zn can lead to diseases such as hyperglycemia and hypercholesterolemia; and excessive Se can cause alopecia, onycholysis, or skin disorders (Yang et al., 2002). In conclusion, excessive metal intake has adverse effects.

### 3.2 Organic Constituents of Pearls

The organic constituents with less than 5% content in pearls (Xia et al., 2010) can be divided into a soluble organic matrix and an insoluble organic matrix (Bédouet et al., 2006; Bédouet et al., 2007; Ma Y. et al., 2011; Ma et al., 2012), which are mainly composed of protein, polypeptide, vitamin B group, porphyrin, and metalloporphyrin compounds. Amongst them, a variety of amino acids can be obtained by protein hydrolysis (pearl hydrolysis), including seven kinds of human essential amino acids (Zhan, 2010; He et al., 2016) (lysine, phenylalanine, methionine, threonine, isoleucine, leucine, and valine) and 10 kinds of non-essential amino acids (aspartate, serine, glutamic acid, proline, glycine, cysteine, alanine, tyrosine, histidine, and arginine) (Bédouet et al., 2007). Studies have also shown that 18 kinds of amino acids can be obtained by hydrolysis from freshwater pearls, including 17 kinds of protein amino acids (including seven kinds of essential amino acids) and a non-protein amino acid (taurine) (Ouyang et al., 2012). Amino acids play an important physiological role in the human body and maintain the body’s normal metabolism. For example, serine, cysteine, and valine can regulate human secretion, enhance immunity, and have an anti-aging effect (He et al., 2016). Glycine can promote the regeneration of skin collagen cells to achieve a cosmetic effect. It can also reduce cholesterol concentration and blood glucose and prevent blood clotting and thrombus. Arginine dilates blood vessels and treats hypertension (Liu and Tian, 2010; He et al., 2016). Aspartate is used medicinally as a cardiological drug and liver function promoter (Liu and Tian, 2010). Glutamate can treat tinnitus, rhinitis, and insomnia (He et al., 2016). The non-protein amino acid taurine can enhance the human body’s metabolism and tranquilize and quiet the spirit (Ouyang et al., 2012). In addition, polypeptides are intermediate products of proteins and have good health care effects (Zheng and Mao, 2004). Vitamin B group also plays an important role in pearls (Zheng and Mao, 2004). Porphyrin and metalloporphyrin compounds, a class of organic substances that can produce various pharmacological effects such as antioxidant activity and improve immunity, play important physiological functions together with proteins in pearls (Zheng and Mao, 2004; Zhan, 2010). The specific functions of various constituents of the organic constituents in pearls are shown in Table 5.

**TABLE 5:** Functions and applications of organic components in pearls.

Composition	Functions	Applications	References
**Serine**	Regulate human secretion, enhance immunity, and antioxidant	Metabolic disorders, low immunity, aging	He et al. (2016)
**Cysteine**	Regulate human secretion, enhance immunity, and antioxidant	Metabolic disorders, low immunity, aging	He et al. (2016)
**Valine**	Regulate human secretion, enhance immunity, and antioxidant	Metabolic disorders, low immunity, aging	He et al. (2016)
**Glycine**	Promote collagen cell regeneration, lower cholesterol concentration and blood sugar level	Cosmesis, hyperglycemia, thrombosis, hemagglutination	He et al. (2016); Liu and Tian. (2010)
**Arginine**	Dilate blood vessels	Hypertension	He et al. (2016)
**Glutamate**	Regulate human secretion, improve memory, calm	Tinnitus, rhinitis, insomnia	He et al. (2016)
**Lysine**	Involved in the synthesis of skeletal muscle, enzymes, and peptide hormones to promote growth and enhance immune function	Anemia, blood ammonia	He et al. (2016)
**Alanine**	Promote the metabolism of alcohol in the blood and enhance liver function	Liver function promoter	Liu and Tian (2010)
**Aspartic acid**	Improve myocardial contractility and enhance liver function	Heart disease, liver function promoter	Liu and Tian (2010)
**Taurine**	Enhance body metabolism, calm	Convulsions, epilepsy	Ouyang et al. (2012); Zheng and Mao. (2004)
**Polypeptide**	Antioxidant, hypoglycemic, hypotensive, hypolipidemic, thrombolytic	Innutrition, low immunity, hyperglycemia, hypertension, hyperlipidemia, cerebral thrombosis	Zhan. (2010); Zheng and Mao (2004)
**Vitamin B group**	Prevent arteriosclerosis, regulate metabolism	Hypertension, scurvy, osteoporosis, oral ulcer	—
**Porphyrin and metalloporphyrin compounds**	Antioxidant, improve immunity	Aging, low immunity	Zhan. (2010); Zheng and Mao (2004)

However, the contents of organic constituents in pearls under different environments are different. Compared with freshwater pearls, the nacre of seawater pearls has a higher content of amino acids; in particular, glycine has the highest content, followed by alanine, aspartate, leucine, and arginine (Zhang et al., 2007a; Zhang et al., 2007b). Zhang et al. (2007b) observed that the total amino acid contents in seawater pearls are not only high but also stable, and the relative content and change trend of each amino acid in a certain range for seawater pearls are basically consistent. However, those in freshwater pearls have more obvious changes, indicating that the medicinal value of seawater pearls is more reliable and stable (Zhang et al., 2007b). However, some studies (Weng, 1989) found that the types and content of freshwater and seawater pearls are basically the same, so freshwater pearls can be used instead of seawater pearls to treat diseases. All in all, seawater and freshwater pearls have their own advantages, and the corresponding pearls or blends should be selected according to the specific conditions of clinical diseases to achieve better curative effects.

Organic constituents are also involved in the formation and color of pearls. In terms of formation, the soluble organic matrix is considered to be an important part of the nucleation and growth of pearl crystals (Bédouet et al., 2006; Ma et al., 2012), including crystal form, nucleation location, crystal size, and morphology (Ma et al., 2012). Additionally, the organic matrix plays a regulatory role in the biomineralization process of pearls, especially controlling the formation of CaCO_3_ crystals (Ouyang, 2019), mainly manifested in organic matrix combination with specific crystal planes, leading to a reduced growth rate in this direction, and these slowly growing planes eventually dominate the morphology of crystals (Ma et al., 2012). A comparative study based on proteomics (Bédouet et al., 2007) determined that certain soluble and insoluble proteins coexist in the nacre, indicating that water-soluble proteins may be the precursors of insoluble protein scaffold in the nacre. In terms of color formation, the main color-causing factors in organic substances are porphyrins and carotenoids, and Raman peaks have been previously detected in natural pearls caused by carotenoids (Ouyang et al., 2012).

## 4 Pharmacological Effects and Partial Mechanism of Action of Pearls

**FIGURE 2:**
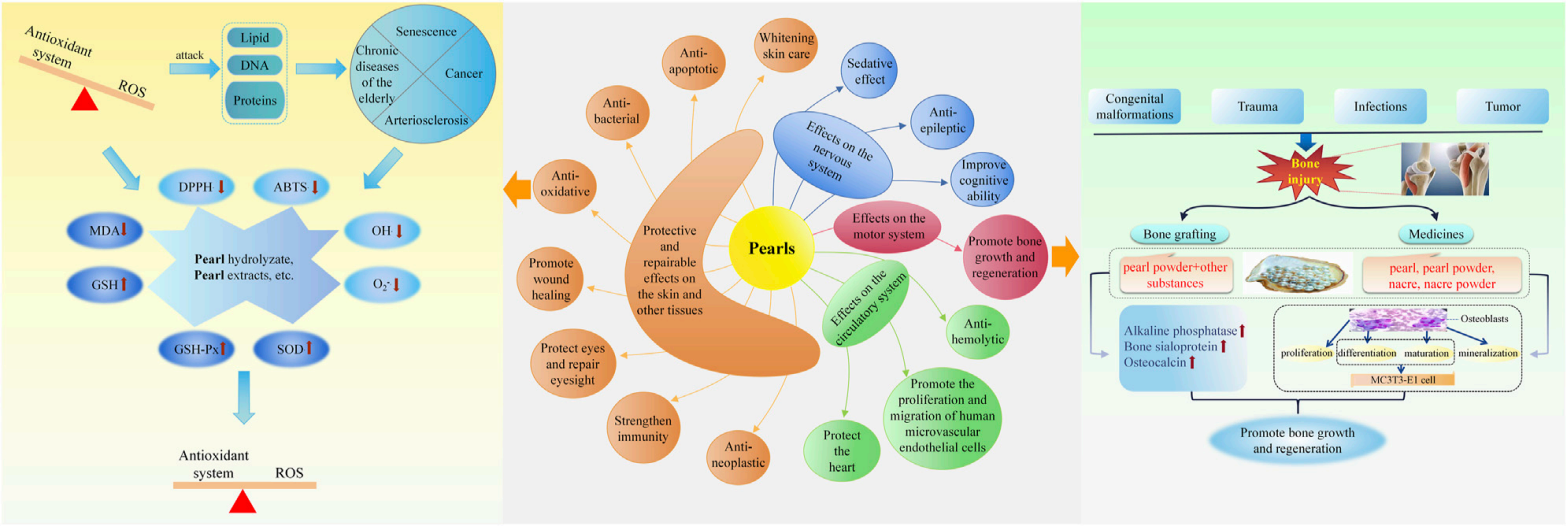
Schematic diagram of the pharmacological effects of pearls and part of the mechanism of action.

By reviewing the literature, we found that pearl powder, nacre powder, pearl extracts, and various preparations of pearls have various pharmacological effects (see Table 6 for details), and this study mainly expounds on their effects on the nervous system, motor system, circulatory system, skin, and other tissues. The schematic diagram of the pharmacological effects of pearls and part of the mechanism of action is shown in Figure 2.

**TABLE 6:** Pharmacological effects of pearls.

Pharmacological effects	Pearls form or preparation	Controls	Animals (Cells) and weight	Model	Dosage (concentration) and mode of administration	Duration of administration	Minimum active dose (concentration)	Results	References
Sedative effect	Pearl powder	①Negative: 0.2% carboxymethyl cellulose solution. ②Negative: 0.2% carboxymethyl cellulose solution	①Rabbit, about 2 kg (half male and half female); ② KM mice, 18–22 g (half male and half female)	① ——. ②Convulsion model in mice induced by caffeine (600 mg/kg)	①500 mg/kg; ip. ②1,200 mg/kg; ip	Once	①500 mg/kg. ②1,200 mg/kg	①The activity of cerebral cortex was inhibited in 83% of rabbits. ②The convulsion latency of mice was prolonged	Pan et al. (1999)
	Nacre powder	Negative: 1% carboxymethyl cellulose solution	KM mice, 18–22 g (half male and half female)	① ——. ②Convulsion model in mice induced by pentylenetetrazol (150 mg kg). ③Sleep model in mice induced by pentobarbital sodium (30 mg/kg)	①Freshwater nacre powder: 600 mg/kg; seawater nacre powder: 600 mg/kg and 1,200 mg/kg; ig. ②Freshwater nacre powder: 600 mg/kg and 1,200 mg/kg; seawater nacre powder: 600 mg/kg and 1,200 mg/kg; ig. ③Freshwater nacre powder: 600 mg/kg and 1,200 mg/kg; seawater nacre powder: 600 mg/kg and 1,200 mg/kg; ig	Once	①Freshwater nacre powder: 600 mg/kg; seawater nacre powder: 1,200 mg/kg. ②Freshwater nacre powder: 600 mg/kg; seawater nacre powder: 600 mg/kg. ③Freshwater nacre powder: 1,200 mg/kg; seawater nacre powder: —	①Decreased spontaneous activity in mice. ②Prolonged convulsion latency in mice. ③Prolonged sleep time in mice	Lu et al. (1991)
	Pearl solution dissolved by enzyme	Negative: distilled water	①KM mice, 18–22 g (male). ②KM mice, 18–22 g (female)	①Pentobarbital sodium (35 mg/kg) induced sleep model in mice. ②Sleep model of mice induced by barbital sodium (0.1 ml/10 g)	4 ml/kg, 8 ml/kg, 12 ml/kg; ig	2 w, once a day	①8 ml/kg. ②8 ml/kg	The spontaneous activity of mice decreased and the sleep time prolonged	Fan (2000)
	Pearl hydrolysate	Negative: normal saline	KM mice, 19–23 g (half male and half female)	——	3 ml/kg, 5 ml/kg; ig	2 d, once a day	3 ml/kg	The spontaneous activity of mice was inhibited	Hu et al. (1994)
	Pearl original powder, pearl water-soluble protein, pearl acid-soluble protein, pearl conchiolin protein, nacre original powder, nacre water-soluble protein, nacre acid-soluble protein and nacre conchiolin protein	Negative: normal saline. Positive: diazepam (2.2 mg/kg)	Mice, 20–22 g (half male and half female)	Convulsion model in mice induced by 0.5% pentylenetetrazole (100 mg/kg)	Pearl original powder (1.1 g/kg), pearl water-soluble protein (0.2 g/kg), pearl acid-soluble protein (0.275 g/kg), pearl conchiolin protein (1.1 g/kg), nacre original powder (1.1 g/kg), nacre water-soluble protein (0.2 g/kg), nacre acid-soluble protein (0.7 g/kg) and nacre conchiolin protein (1.1 g/kg); ig	3 d, once a day	Pearl original powder: 1.1 g/kg. Pearl water-soluble protein: 0.2 g/kg. Pearl acid-soluble protein: 0.275 g/kg. Pearl conchiolin protein: 1.1 g/kg. Nacre original powder: 1.1 g/kg. Nacre water-soluble protein: 0.2 g/kg. Nacre acid-soluble protein: 0.7 g/kg nacre conchiolin protein: 1.1 g/kg	Compared with the control group, diazepam, pearl conchiolin protein, nacre conchiolin protein are very significantly reduced crossing behaviors in mice, diazepam, pearl water-soluble protein, pearl conchiolin protein, nacre original powder, nacre acid-soluble protein, and nacre conchiolin protein are very significantly reduced rearing behaviors in mice; except for nacre acid-solid protein, the others can increase the convulsion latency of mice	Zhang et al. (2016)
	Qishiwei Zhenzhu pills	①Negative: tap water. ②Negative: tap water. Positive: diazepam injection (0.002 g/kg). ③Negative: tap water	KM Mice, 18–24 g (half male and half female)	①——. ②Sleep model in mice induced by pentobarbital sodium (30 mg/kg). ③Sleep model of mice induced by ether (40 ml)	①1 g/kg, 2 g/kg; ig ②1 g/kg, 2 g/kg, 4 g/kg; ig ③0.25 g/kg, 0.5 g/kg, 1.0 g/kg; ig	①Once. ②3 d, once a day. ③3 d, once a day	①1 g/kg. ②1 g/kg. ③0.5 g/kg	①The activity of mice was inhibited. ②All three doses could prolong the sleep time of mice, and the effect decreased at 4 g/kg. ③The doses of 0.5 g/kg and 1.0 g/kg could prolong the sleep time of mice	Wen et al. (1998)
	Improve cognitive ability	Pearl powder	Negative: normal saline. Positive: estazolam (0.13 mg/kg)	SD rats, 160–200 g (male)	Sleep deprivation model	2.5 mg/kg; ig	14 d, once a day	2.5 mg/kg	Pearl powder and estazolam can significantly improve the loss of spatial learning and memory caused by sleep deprivation stress	Xia et al. (2020)
	Anti-epileptic effect	Pearl original powder, pearl water-soluble protein, pearl acid-soluble protein, pearl conchiolin protein, nacre original powder, nacre water-soluble protein, nacre acid-soluble protein and nacre conchiolin protein	Negative: normal saline. Positive: diazepam (2.2 mg/kg)	Mice, 20–22 g (half male and half female)	Convulsion model in mice induced by 0.5% pentylenetetrazole (100 mg/kg)	Pearl original powder (1.1 g/kg), pearl water-soluble protein (0.2 g/kg), pearl acid-soluble protein (0.275 g/kg), pearl conchiolin protein (1.1 g/kg), nacre original powder (1.1 g/kg), nacre water-soluble protein (0.2 g/kg), nacre acid-soluble protein (0.7 g/kg) and nacre conchiolin protein (1.1 g/kg); ig	3 d, once a day	Pearl acid-soluble protein: 0.275 g/kg. Nacre original powder: 1.1 g/kg. Nacre water-soluble protein: 0.2 g/kg. Nacre acid-soluble protein: 0.7 g/kg. Nacre conchiolin protein: 1.1 g/kg	5-HT3↓, GABAB↑. The convulsion latency of mice increased	Zhang et al. (2016)
	Promote bone growth and regeneration	Pearl	①Shell nacre ②hydroxyapatite (HA)	①Simulated body fluid (SPF). ②OCT-1 cells	*In vitro*	①Pearl soaked in SPF. ②OCT-1 cells were cultured on pearl specimens	①1 w, medium was changed every 2 days. ②5 d, medium was changed every 2 days	——	①HA particles were rapidly formed on the surface of pearls soaked in SPF. ②Pearl can promote the proliferation of osteoblasts, and the proliferation speed is faster and more stable	Shen et al. (2006)
		Water-soluble nano-pearl powder (WSNPP)	——	MC3T3-E1 cells	*In vitro*	WSNNP protein: 0 μg/ml, 10 μg/ml, 25 μg/ml and 50 μg/ml; the above four doses of WSNNP protein were added to MC3T3-E1 cells for culture	48 h	10 μg/ml WSNNP protein	10 μg/ml, 25 μg/ml, 50 μg/ml WSNNP can stimulate the viability of MC3T3-E1 cells, and 50 μg/ml WSNNP has the greatest effect (collagen I↑, SPP1↑, RUNX2↑); WSNNP promotes the proliferation of MC3T3-E1 cells through autophagy (LC3II/I↑, Beclin1↑ and ATG7↑)	Cheng et al. (2018)
		Freshwater pearl powder	Positive: nano-hydroxyapatite	Rabbit, 2.5–3 kg	Bone defect model	Nano-freshwater pearl powder and micro- freshwater pearl powder were implanted into the rabbit model of bone defect, respectively	——	——	The recovery degree of bone defect was the best for nano-hydroxyapatite, followed by nano-freshwater pearl powder	Chen et al. (2017)
Nacre		——	Human bone cells (HBCs)	*In vitro*	Cells culture	1, 2, 3, 4 w, medium was changed once daily	——	Promote gene expression of alkaline phosphatase, bone sialoprotein, and osteocalcin	Pattapon et al. (2011)
Nacre water-soluble organic matrix (WSM), four components from WSM: SE1 –SE4		Positive: recombinant human BMP-2 (rhBMP-2) (12.5–50 ng/ml), dexamethasone (Dex) (1 µM)	MRC5 human fetal lung fibroblasts, bone marrow cells, osteoblasts	*In vitro*	WSM protein: 81–1,300 μg/ml; cells culture	MRC5 human fetal lung fibroblasts: 3–13 d, medium was changed two times per week bone marrow cells: medium was changed every 3 days. Osteoblasts: —	WSM protein: 325 μg/ml	WSM stimulated alkaline phosphatase (ALP) activity in a dose-dependent manner, when cultured with SE4, ALP↓	Mouriès et al. (2002)
①Nacre powder. ②Nacre powder water-soluble matrix (WSM)		——	①Sheep ②Bone marrow	①*In vivo*: cavity model. ②*In vitro*	①Nacre powder was implanted into the cavity model. ②WSM protein: 0–2,000 μg/ml; cells culture	①1, 8, 12 w ②7 d, medium was changed every 3 days	——	①There was no inflammation and foreign body reaction at 1 w, the blood vessels began to regenerate at 8 w and new bone appeared in the cavity at 12 w, and the new bone was in contact with or adjacent to the nacre. ②ALP↑	Lamghari et al. (1999)
Nacre water-soluble matrix		——	Preosteoblast (MC3T3-E1) cells	*In vitro*	0.005% w/v, 0.025% w/v, and 0.05% w/v; cells culture	24 h	0.025% w/v	ALP↑, osteocalcin. (OCN)↑, pro-alpha 2(I) collagen (COL-1A2)↑, osteoblast differentiation is promoted	Chaturvedi et al. (2013)
Poly-l-lactide (PLLA)/aragonite pearl powder. PLLA/vaterite pearl powder and PLLA/nacre powder scaffolds		PLLA scaffolds	Rat bone marrow-derived mesenchymal stem cells (rBMSC)	*In vitro*	PLLA/powder: 80/20; cells culture, the cells were seeded onto the scaffold	1, 3, 7 d, medium was changed every 3 days	PLLA/powder: 80/20	PLLA, PLLA/aragonite and PLLA/nacre have good cell compatibility and promote the attachment and growth of cells on the scaffold surface and pores; compared with the control group, PLLA/aragonite and PLLA/nacre significantly promoted proliferation and differentiation of rBMSC cells	Liu et al. (2013)
Polylactic acid (PLA)/pearl powder scaffolds		PLA scaffolds, PLA/CaCO3 (80/20) scaffolds	MC3T3 cells	*In vitro*	Pearl powder/PLA: 0%, 5%, 10%, 15%, and 20%; cells culture, the cells were seeded onto the composites and PLA	1, 4, 7 d, medium was changed every 2 days	Pearl powder/PLA: 5%	PLA/pearl powder scaffold has better ability to promote cell proliferation and good cell morphology than the original PLA	Dai et al. (2015)
Nano-nacre/type I collagen scaffolds		type I collagen scaffolds	MC3T3-E1 cells	*In vitro*	Nano-nacre/type I collagen: 2/1; cells culture, the cells were seeded onto the scaffold	1, 4, 7 d, medium was changed every 2 days	Nano-nacre/type I collagen: 2/1	Compared with the control group, nano-nacre/type I collagen scaffold can significantly promote the adhesion, proliferation, and differentiation of MC3T3-E1 cells, among them, ALP↑ and Col-1↑	Xu et al. (2014a)
Nano-pearl powder/chitosan hyaluronic acid scaffolds (NPP/C-HA)		NPP scaffolds, C-HA scaffolds	Rabbit	Rabbit distal femoral bone defect model	Nano-pearl powder/chitosan/hyaluronic acid: 10/4/1; different materials were implanted into the bone defect area of rabbits	The implants were stored for 4, 8, and 12 w after implantation	Nano-pearl powder/chitosan/hyaluronic acid: 10/4/1	NPP/C-HA scaffold has good biocompatibility and bone promoting effect	Wang et al. (2019)
Nano-pearl powder/recombinant human bone morphogenetic protein 2 (rhBMP-2)/hyaluronic acid (HA)		Nano-pearl powder/hyaluronic acid (10/1), nano-pearl powder	Rabbit, 2–2.5 kg (male)	Rabbit distal femoral bone defect model	Nano-pearl powder/rhBMP-2/HA: 200,000/1/40,000; different materials were implanted into the bone defect area of rabbits	The implants were stored for 4, 8, and 12 w after implantation	Nano-pearl powder/rhBMP-2/HA: 200,000/1/40,000	Nano-pearl powder and artificial bone can promote the repair of bone defects, and nano-pearl powder artificial bone containing rhBMP-2 has a better effect on the osteogenic repair of bone defects	Li et al. (2020)
Dialdehyde bletilla striata glucomanna/hydroxypropyl chitosan/nano nacre powder scaffolds (DBsGM/HPCS/NNP)		——	Wistar rats, 150–180 g (male)	Rat mandibular defect model	The material was implanted into the bone defect area of rats	The implants were stored for 2, 4, 6, and 8 w after implantation	——	DBsGM/HPCS/NNP composite scaffold has good osteogenic effect in rat mandibular defect	Chen et al. (2016)
Nacre powder/platelet-rich fibrin (NP/PRF)		①Nacre powder water-soluble matrix (WSM), 10% PRF. ②NP, PRF	①MC3T3-E1 cells. ②Rabbit, 2.1–2.7 kg	①*In vitro*. ②*In vivo*: rabbit skull defect model	①10% PRF/nacre powder WSM (200 μg/ml): 1/2; cells culture. ②NP/PRF: 1/2; different materials were implanted into the defect area of rabbit skull	①3, 5 d, medium was changed every 1 day. ②The implants were stored for 4, 8, and 12 w after implantation	①10% PRF/nacre powder WSM (200 μg/ml): 1/2. ②NP/PRF: 1/2	①NP/PRF is conducive to the proliferation of MC3T3-E1 cells, among them ALP↑. ②The mixed material group has obvious osteogenic effect and can promote vascular regeneration	Huang (2020)
Protect the heart		Water-soluble pearl powder and ordinary pearl powder	Negative: water	Wistar rats, 180–205 g (male or female)	Aconitine (about 0.0 L 6 L m L/m in) was injected intravenously at a constant rate to the arrhythmia model	Water-soluble pearl powder: 0.5 g/kg, 1 g/kg; ig. Ordinary pearl powder: 0.5 g/k g, 1 g/kg; ig	Once	Water-soluble pearl powder: 1 g/kg. Ordinary pearl powder —	Water-soluble pearl powder (1 g/kg) can significantly accelerate the stable recovery of sinus rhythm	Zhang et al. (1994)
Promote the proliferation and migration of human microvascular endothelial cells		Pearl hydrolysate	——	Human microvascular endothelial cells	*In vitro*	120, 60, 30 mg/L; cells culture	The medium was changed every 2 days	30 mg/L	Pearl hydrolysate cultured cells had stronger proliferation ability and increased the number of migrating cells	Cen et al. (2018)
Anti-haemolytic effect		Pearl powder		Erythrocytes	*In vitro*: 2,2′-Azobis (2-amidinopropane) dihydrochloride (AAPH) induced hemolysis	50–200 μg/ml; cells culture	2, 4, 6 h	50 μg/ml	Different concentrations of pearl powder (50–200 μg/ml) have significant inhibitory effects on the pre incubation of red blood cells	Yang et al. (2017)
Anti-oxidative effect		Seawater pearl hydrolysate	Positive: vitamin C	DPPH·, ABTS·, OH·, O2-·	*In vitro*	2 , 5 , 5, and 0.2 ml of seawater pearl hydrolysate with different volume fractions (0%, 4%, 8%, 12%, 16%, 20%) were added to DPPH·, OH·, O2-· and ABTS ·, respectively	——	4% seawater pearl hydrolysate	Seawater pearl hydrolysate has a strong scavenging effect on DPPH· and ABTS· whereas its scavenging ability on O2-· and OH· is relatively weak	(Pu et al., 2017; Pu et al., 2018a)
		Pearl powder, protein extract in pearl powder, and non-protein extract in pearl powder	——	DPPH·, O2-·, *C. elegans*	*In vitro*	5–100 mg/ml; three different pearl types were added to DPPH · and O2- · respectively; culturing of *C. elegans*	——	DPPH·, O2-·: 5 mg/ml. Culturing of *C. elegans*.: 10 mg/ml	Three different types of pearls can effectively remove DPPH· and O2-·, and prolong the life of *C. elegans*, especially the function of protein extract in pearl powder	Chiu et al. (2018)
		Water-soluble pearl powder and purified pearl protein	——	DPPH·, OH·, O2-·	*In vitro*	Water-soluble pearl powder: 5, 10, 15 , 20 , 25 mg/ml, added to DPPH·, OH· and O2-·, respectively. Purified pearl protein: 0.1 , 0.2 , 0.3 , 0.4 , 0.5 mg/ml, added to DPPH·, OH· and O2-·, respectively	——	OH·: water soluble pearl powder 5 mg/ml, purified pearl protein 0.1 mg/ml. DPPH· and O2-·: —	Water-soluble pearl powder and purified pearl protein did not have obvious scavenging effect on DPPH· and O2-· but had strong scavenging ability on OH·	Liao (2019)
		Freshwater pearl extracts	DPPH·: negative-absolute ethanol, positive-vitamin C. O2-·: negative- distilled water, positive-vitamin C	DPPH·, O2-·	*In vitro*	0, 5, 10, 15, 20 μg/ml, added to DPPH· and O2-·, respectively	——	5 μg/ml	Pearl extract has high antioxidant capacity and the ability to scavenge free radicals instead of SOD enzyme, 2 μg/ml pearl extract is equivalent to (83.7 ± 1.2) U/ml SOD enzyme	Yang et al. (2015a)
		Pearl hydrolysate	Negative: normal saline	Aging rats, 290–340 g (half male and half female). Youth rats, 168–192 g (half male and half female)	——	3, 2 ml/kg; ig	23 d, once a day	2 ml/kg	Pearl hydrolysate can increase SOD activity and reduce lipofuscin content	Hu et al. (1994)
		Pearl hydrolysate	Negative: normal saline	HLE-B3 cells	*In vitro*: HLE-B3 cells were treated with 200 μmol/L H2O2 to oxidative damage	0, 50, 100, 200 mg/L; cells culture	24, 48 h	50 mg/L	GSH-Px↑, SOD↑, MDA↓	Liu et al. (2020a)
		Pearl hydrolysate	Negative: normal saline	HMEC -1 cells	*In vitro*: HMEC -1 cells was treated with 200 μmol/L H2O2 to oxidative damage	30 mg/L, 60 mg/L, 120 mg/L; cells culture	24 h	30 mg/L	GSH-Px↑, SOD↑, MDA↓, ROS↓, at 120 mg/L, LC3-Ⅰ/LC3-Ⅱ↓	Liu et al. (2020b)
		Strengthen immunity	Pearl powder	Negative: normal saline (2 ml/kg). Positive: ginseng powder (78 mg/kg)	Wistar rats, 200–250 g (male)	——	260 mg/kg; ig	10 d, once a day	260 mg/kg	The ratio of T lymphocytes in peripheral blood and the ratio of antibody forming cells in spleen increased significantly; the phagocytosis of neutrophils in peripheral blood was significantly enhanced	Wang et al. (1994)
			Pearl powder	Negative: distilled water	KM mice, 18–22 g (female)	——	0.33, 0.67, 2 g/kg; ig	30 d, once a day	0.33 g/kg	The level of serum hemolysin and the activity of natural killer cells (NK cells) were increased, and the cellular immunity and phagocytosis were enhanced	Qian and Zhu (2003)
	Hydrolyzed pearl tablets		Negative: distilled water Positive: lentinan tablets	KM mice	Immunocompromised model in mice induced by cyclophosphamide	2.04 , 1.02, 0.51 g/kg; ig	30 d, once a day	0.51 g/kg	The carbon clearance index K of monocyte macrophage system and the number of peripheral blood T lymphocytes were increased, and the production of serum hemolysin was promoted	Lan et al. (2017)
	Hydrolyzed seawater pearl tablet (HSPT)		Negative: normal saline. Positive: N-acetylcysteine (NAC, 150 mg/kg)	C57BL/6 mice (female)	Mice model of chronic obstructive pulmonary disease induced by smoking	ig	7 w, dosing started at 3 W until end	——	IFN-c↓, IL-2↓, IL-4↓, IL-10↓; the proportion of spleen CD3+/CD4+ T lymphocytes was reduced	Chen et al. (2020)
	Pearl in ashed form		Negative: 0.5% sodium carboxymethyl cellulose solution	Swiss mice, 20–25 g (male)	——	25, 50, 100, 500 μg/kg; ig	10 d, once a day	25 μg/kg	IgG↑, IgG1↑, IgG2a↑ and IgG2b↑, the effect of 50 μg/kg was the most significant	Elahi et al. (2014)
Pearl pulvis	Negative: normal saline (20 ml/kg). Positive: levamisole (25 mg/kg)		BALB/C mice, 18–22 g (half male and half female)	Immunocompromised model	Low dose: 2.5 g/kg; apply on the back once a day. Medium dose: 2.5 g/kg; apply on the back twice a day. High dose: 2.5 g/kg; apply on the back three times a day	10 d	Low dose	The phagocytosis of macrophages, the production of serum hemolysin, and the proliferation of T lymphocytes in immunocompromised mice were enhanced	Liu et al. (2003)
Promote wound healing	Pearl extract gel		——	Human keratinocyte HaCaT cells	*In vitro*: ultraviolet B (UVB) radiation cells	0–100 μg/ml, cell culture	All experiments were performed after 10 passages	——	The cellular inflammation caused by UVB irradiation was repaired	Yang et al. (2015b)
	Nacre powder		Negative: distilled water	SD rats, 160–200 g (half male and half female)	1.5 ml rat gastric ulcer model induced by hydrochloric acid ethanol solution	8, 4, 2, 1, 0.5 g/kg; ig	Once	0.5 g/kg	The anti-ulcer effect of ultra-fine nacre powder is better than that of nacre powder, but both have anti-ulcer effect	Xia et al. (2014)
	Zhushen pulvis		①Negative: distilled water. Positive: aspirin (300 mg/kg). ②Negative: distilled water. ③Negative: distilled water. Positive: dexamethasone (2 mg/kg). ④ Negative: distilled water. Positive: aspirin (300 mg/kg)	①KM mice (half male and half female). ②KM mice (half male and half female). ③Wister rats, 80–120 g (half male and half female). ④Wister rats, 120–160 g (male)	①Mice auricle swelling model induced by xylene. ②Histamine induced capillary permeability enhancement model in mice. ③Rat granuloma model. ④Rat foot swelling model induced by egg white	0.5, 0.25, 0.1 ml/kg; ig	7 d, once a day	0.1 ml/kg	①Mice auricle swelling was significantly inhibited. ②Decreased capillary permeability in mice. ③Granuloma in rats was significantly improved. ④Rat foot swelling was inhibited	Lin et al. (1996)
	Zhuhuang ointments		①Negative: normal saline. ②Negative: normal saline. Positive: acetic acid skin relaxing ointments (0.01 g)	①Mice, 20–26 g (half male and half female). ②Mice, 20–26 g (male)	①Ⅱ degree scald model. ②Mice ear inflammation model induced by 2% croton oil	①0.5 g; apply it to the affected area. ②0.01 g; apply it to the affected area	②17 d, twice a day. ②1 day, three times in total	①0.5 g. ②0.01 g	①It has a certain therapeutic effect on Ⅱ degree scald in mice. ②Zhuhuang ointments have better anti-inflammatory effect	Liu et al. (1995)
	Zhenzhu Shaoshang ointments		Negative: matrix. Positive: sulfadiazine silver	Rabbit	Ⅲ degree burn model induced by hydrothermal solution, hot iron sheet, and concentrated sulfuric acid	0.5 g, apply it to the affected area	7, 14, 28 d, once a day	0.5 g	Compared with the control group, Zhenzhu Shaoshang ointments can significantly improve the burn caused by hot liquid and hot iron sheet. At 14 days, the burn area is significantly reduced; at 28 days, the scar area decreased significantly. It has no obvious effect on burns caused by concentrated sulfuric acid	Huang et al. (1997)
	Zhenzhu pulvis	Negative: normal saline. Positive: arnebia oil	ICR mice, 20–30 g (half male and half female)	Ⅱ degree scald model	Wrap the medicine with gauze and apply it to the affected area	4, 7, 10, 14 d, change the dressing once a day	——	The wound healing rate of the experimental group was better, among them bFGF↑	Kan et al. (2015)
	Whitening skin care	Water-soluble pearl powder and purified pearl protein	Positive: arbutin (0.25–3%)	Tyrosinase	*In vitro*	Water-soluble pearl powder: 5 , 10 , 15 , 20 , 25 g/ml; adding drugs to tyrosinase purified pearl protein: 0.00312 , 0.00625, 0.0125, 0.025, 0.05, 0.1, 0.2 mg/ml; adding drugs to tyrosinase	——	Water-soluble pearl powder: 5 g/ml purified pearl protein: 0.00312 mg/ml	The inhibition rate of tyrosinase increased with the increase in sample concentration. When the concentration of purified pearl protein was 0.0125 mg/ml, the inhibition rate was equivalent to that of 1% arbutin	Liao (2019)
		Enzymatic pearl extracts	Positive: arbutin (10 mg/L), fruit acid (10 mg/L)	B16 melanoma cells	*In vitro*	10, 20, 40, 80, 100 mg/L; cell culture	3 h	10 mg/L	Pearl extract reduced melanin content and tyrosinase activity without affecting cell growth base, while positive drugs showed an upward trend or no obvious decrease	(Yang et al., 2016a; Yang et al., 2018a)
		Pearl extracts (conventional solid-liquid extraction, ultrasonic assisted extraction, microwave assisted extraction, acid hydrolysis and enzymatic hydrolysis)	Positive: kojic acid (0.013, 0.025, 0.05, 0.1, 0.2 mg/ml)	B16F10 cells	*In vitro*	Conventional solid-liquid extraction, ultrasonic assisted extraction and microwave assisted extraction: 0.063, 0.125, 0.25, 0.5, 1 g/ml. Acid hydrolysis and enzymatic hydrolysis: 0.006, 0.013, 0.025, 0.05, 0.1 g/ml; cell culture	24 h	Conventional solid-liquid extraction, ultrasonic-assisted extraction, and microwave-assisted extraction: 0.063 g/ml. Acid hydrolysis and enzymatic hydrolysis: 0.006 g/ml	The inhibition rate of tyrosinase increased with the increase of concentration, among which the inhibition of microwave extracts was the most significant, and the enzymatic hydrolysis extracts were equivalent	Shen (2017)
		Pearl mixture dissolved by acid and enzyme	Positive: arbutin (0.1%)	Human melanocytes	*In vitro*	0.25%, 0.5%, 0.75%; cell culture	48 h	0.25%	The difference between positive drugs and 0.75% concentration of pearl hydrolysate was very significant, and the other differences were only significant	Deng et al. (2014)
		Acid hydrolyzed pearl solution	Positive: arbutin	Mice B16 melanoma cells	*In vitro*	2.5%, 5%, 7.5%, 10%, 15%, 20%, 25%; cell culture	72 h	2.50%	The inhibition rate of tyrosinase increased with the increase of concentration	Pu and Tong (2015)
Protect eyes and repair eyesight		Pearl powder	Positive: 0.25% chloramphenicol eye drops	Rabbit, 2.46–3.91 kg (male)	Rabbit corneal injury model	50 mg; dot eyes with pearl powder	11–30 d, three times a day	50 mg	The ulcer healed, the scar decreased, and the thickness of corneal pannus decreased. The effect of the experimental group was better	Lu et al. (1990)
		Nacre powder hydrolysate	Positive: zinc gluconate eye drops (zinc containing: 0.3 and 0.15 mg/ml)	Rabbit	——	Nitrogen content: 12.29 mg/100 ml, 6.417 mg/100 ml; 0.1 ml/time; drop eye	10 d, three times a day	Nitrogen content: 6.417 mg/100 ml	Promote eye microcirculation and speed up blood flow	Gao et al. (2000)
		Fufang Zhenzhu hydrolysate	Negative: normal saline	Chicken	Form deprivation myopia model	Drop eye	5 w, four times a day	——	Fufang Zhenzhu hydrolysate can inhibit the expansion of the outer diameter, inner diameter, and equatorial radius of the eyeball	Chen et al. (2001)
		Zhenzhu pills	Positive: vitamin E(1 mg/kg)	Rabbit, 2.5–3 kg (half male and half female)	Retinal ischemia-reperfusion model	0.15 g/kg; ig	7 d, once a day	0.15 g/kg	Protect retina from ischemia-reperfusion injury	Meng et al. (2007)
	Anti-apoptotic effect	Porphyrin compounds from pearl powder	①Negative: normal saline. ②——	①Mice, 18–22 g (half male and half female). ② P388/J3 cells	①*In vivo*: S180 sarcoma cells were inoculated subcutaneously into the right armpit of mice. ②*In vitro*	①40 mg/kg; ip. ②50 μg/ml, 100 μg/ml; cell culture	①9 d, once a day. ②0–96 h	①40 mg/kg. ②50 μg/ml	①The inhibition rate of S180 sarcoma cells was 34.8%. ②It has a certain killing effect on P388/J3 cells cultured *in vitro*	Chen and Yuan (1990)
	Anti-apoptotic effect	Hydrolyzed pearl extracts	Positive: vitamin C (5.68 mmol/L)	Fibroblast	*In vitro*: oxidative damage of cells by H2O2	Protein content: 16.94 and 169.4 μg/ml; cell culture	4 h	Protein content: 16.94 μg/ml	Pearl extracts can inhibit the damage and apoptosis of human skin fibroblasts mediated by hydrogen peroxide to a certain extent, and the cell survival rate increased significantly	Wang et al. (2018)
		Enzymatic pearl extracts	——	Human epidermal keratinocytes	*In vitro*: UV induced cell damage	Protein content: 0.21% and 2.1%; cell culture	24 h	Protein content: 0.21%	Pearl extracts can inhibit UV mediated damage and apoptosis of human keratinocytes to a certain extent, and the cell survival rate increased significantly	Yang et al. (2018b)
Anti-apoptotic effect		Acid soluble pearl extracts	Negative: sterile water	*Staphylococcus aureus*	*In vitro*	240, 120, 60, 30, 15, 7.5, 3.75, 1.88, 0.94 , 0.47, 0.24 mg/ml; bacterial culture	24 h	0.94 mg/ml	The bacteriostatic circle reached 17.861 ± 0.948 mm, and the bacteriostatic activity was hardly affected by the outside world	Liu et al. (2020)

Note: “w" means “week”, “d" means “day”, “h" means “hour”, “ig” means “intragastric administration”, and “ip” means “intraperitoneal administration".

### 4.1 Effects on the Nervous System

#### 4.1.1 Sedative Effect

The theory of traditional Chinese medicine believes that the properties of pearls are heavy, sinking, and descending. The property of heavy can be used to suppress timidity and restlessness, so pearls have the effect of calming the nerves and convulsions, treating the disquieted mind, heart palpitations, and insomnia (Zhao, 2015).

In modern scientific research, pearl powder (Pan et al., 1999; Zhang et al., 2016), nacre powder (Lu et al., 1991), pearl solution dissolved by enzyme and acid-base (Hu et al., 1994; Fan, 2000), pearl protein extracts (Zhang et al., 2016) and pearls’ compound preparation Qishiwei Zhenzhu pills (Wen et al., 1998) have a good sedative effect. They achieve a sedative effect by reducing the activity of the cerebral cortex and inhibiting the action of the central nervous system, which are mainly manifested in the inhibition of the spontaneous activity of animals, the prolongation of convulsion latency, the improvement of sleep, and the synergy of pentobarbital sodium or ether to prolong sleep time. In the comparative study of pearl powder and pearl protein extracts, original pearl powder and pearl conchiolin protein most significantly inhibited the activity of mice (Zhang et al., 2016), suggesting that pearl conchiolin protein may be the key constituent of the sedative effect. In addition, the sedative effect of the compound preparation of Qishiwei Zhenzhu pills may be related to the efficacy of pearls, but due to the large number of medicinal materials contained in the preparation, the role of pearls in this preparation still needs to be explored.

#### 4.1.2 Improve Cognitive Ability

Sleep deprivation can mimic insomnia by altering the expression of hippocampal proteins, which in turn can cause cognitive decline. The Morris water maze test confirmed that pearl powder could improve the spatial learning and memory decline induced by sleep deprivation in rats, and the low expression of three hippocampal proteins (RIMS3, Ppp1r14a, and MGR3) was reversed by pearl powder during the process. This indicates that pearl powder can significantly ameliorate hippocampal injury in rats’ brains and improve cognitive ability (Xia et al., 2020).

#### 4.1.3 Anti-Epileptic Effect

Different types of seizures are associated with the intracerebral excitatory neurotransmitter 5-hydroxytryptamine (5-HT) and inhibitory neurotransmitter γ-aminobutyric acid (GABA). After epileptic seizures were induced by pentylenetetrazole, the expression of 5-HT3 was upregulated, and the level of GABA_B_ was downregulated. After treatment with the original pearl powder, pearl water-soluble protein, pearl acid-soluble protein, and pearl conchiolin protein, the expression of 5-HT3 and the level of GABA_B_ were recovered to some extent, and the protein extracts exhibited the most significant effect (Zhang et al., 2016). This indicates that proteins may be the active constituents exerting the anti-epileptic effect.

### 4.2 Effects on the Motor System

The motor system contains bone, joints, and skeletal muscle. The role of the bone in the motor system is indispensable. Bone defects are one of the common phenomena in clinics, and their formation is related to many factors, such as congenital malformations, trauma, infection, tumor, and pathological factors, which are difficult to treat. Human intervention is required to promote bone tissue regeneration when the loss is too great, or the self-repair ability declines. Bone grafting is the main way to solve this problem, and the choice of bone grafting materials becomes the key to good or bad bone repair (Mao and Xu, 2016; Li et al., 2018; Wang et al., 2019). Therefore, high biological activity, bio-compatibility, osteoconduction, and biodegradability have become necessary conditions for bone grafting materials (Liu et al., 2013; Cheng et al., 2018). General bone injuries can be treated with drugs, such as pro-bone forming and bone resorption inhibiting drugs (Chen et al., 2004; Wang et al., 2014).

Pearls used in traditional medicine have played an important role in bone injury as a bone-promoting medicine and a good bone grafting material. In terms of promoting bone formation, some studies (Shen et al., 2006) used the shell nacre and hyaluronic acid as control materials, soaked pearls in simulated body fluids, and conducted cell culture to evaluate the osteogenic activity of pearls. The results showed that pearls could stimulate osteoblast proliferation, which is faster and more stable than shell nacre and hyaluronic acid. However, most pearls are ground into powder clinically for bone injury treatment because the physical arrangement of crystals in pearl powder greatly enhances the ability of osteoinduction (Cheng et al., 2018), and the chemical constituents of general and nano-pearl powder are more easily utilized by the human body. Compared with general pearl powder, nano-pearl powder shows better curative efficacy. An *in vivo* experiment to repair bone defects of the distal femur of rabbits found that nano-pearl powder is superior to micron-size pearl powder in the percentage of the area of newly forming bone tissue, degradation speed, and repair ability, indicating that nano-pearl powder has a stronger ability to restore bone tissue (Chen et al., 2017). Of course, the nacre also has the same osteogenic effect. Pattapon et al. (2011) studied the role of the nacre in inducing bone regeneration through the gene expression of bone markers (alkaline phosphatase, bone sialoprotein, and osteocalcin) and the production of bone sialoprotein (Pattapon et al., 2011). They found that the nacre can promote the expression and production of these bone markers. The good cell biocompatibility of the nacre contributes to its excellent bone repair effect (Liu et al., 2014). Nacre powder has a more significant effect because of its small particle size (Atlan et al., 1997). In addition, pearl water-soluble organic matrix or nacre water-soluble organic matrix has a good osteogenic effect, which is specifically manifested as they can push the differentiation and maturation of fibroblasts, bone marrow stromal cells, and osteoblasts, prolonging the life span of bone cells. (Lamghari et al., 1999; Mouriès et al., 2002; Oliveira et al., 2012; Chaturvedi et al., 2013). The mechanisms of the above osteogenesis can be summarized as follows: firstly, the differentiation of MC3T3-E1 cells is promoted by enhancing autophagy; secondly, autophagy in MC3T3-E1 cells is simulated through the MEK/ERK signaling pathway (Wang et al., 2014; Cheng et al., 2018). In conclusion, MC3T3-E1 cells are indispensable during osteogenesis.

In terms of bone grafting materials, pearls in the form of powder are usually combined with other substances to form composite materials as bone substitutes and promote osteogenesis. For example, polylactic acid (PLA)/pearl or nacre powder scaffolds have two times higher compressive strength than PLA scaffolds alone and have a significantly stronger promoting effect on the proliferation and alkaline phosphatase activity of bone marrow mesenchymal stem cells than PLA (Liu et al., 2013). Dai et al. (2015) also showed that adding pearls to PLA helps the deposition of hydroxyapatite and accelerates the proliferation of MC3T3-E1 cells, which is a better bone repair material (Dai et al., 2015). Another example is the nano-nacre/type I collagen composite scaffolds, which can also promote the growth of MC3T3-E1 cells and increase the related bone marker alkaline phosphatase activity and collagen expression level (Xu J. H. et al., 2014). In addition, nano-pearl powder/chitosan hyaluronic acid scaffolds (Wang et al., 2019) and nano-pearl powder/rhBMP-2/hyaluronic acid composite materials (Li et al., 2020) can better repair bone defects in rabbit distal femurs. Dialdehyde bletilla striata glucomanna/hydroxypropyl chitosan/nano-nacre powder scaffolds can also promote bone formation in rat mandibular defects (Chen et al., 2016). Similarly, the composite composed of nacre powder/platelet-rich fibrin has better osteogenic activity than nacre powder and platelet-rich fibrin alone (Huang, 2020). The above bone repair composite materials have the characteristics of good biocompatibility, biological activity, and good three-dimensional structure. The existence of pearls not only lays a more solid foundation for the properties of composites but also provides better conditions for long-term cell proliferation in the osteogenic activity of some materials (Dai et al., 2015).

### 4.3 Effects on the Circulatory System

#### 4.3.1 Protect the Heart

Water-soluble pearl powder exhibits cardioprotective effects, mainly in terms of its ability to improve cardiac contractility and accelerate the recovery of sinus rhythm, and it can exhibit certain antiarrhythmic (aconitine triggered) effects after multiple administrations of larger doses of water-soluble pearl powder (1 g/kg). However, ordinary pearl powder has no obvious effect, which may be related to its solubility in water, because the solubility of ordinary pearl powder is not good, and the active constituents are not easily volatilized (Zhang et al., 1994). Therefore, the extraction of the active constituents of pearls in the later stage is also the key point of research.

#### 4.3.2 Promote the Proliferation and Migration of Human Microvascular Endothelial Cells

Vascular endothelial cell dysfunction plays an important role in the occurrence and development of hypertension, and improving vascular endothelial cell function has become an important measure for the treatment of hypertension. A study found that the use of pearl hydrolysate to culture human microvascular endothelial cells can significantly promote their division and proliferation, and the higher the concentration, the stronger the promoting effect. The number of migrating cells also increased significantly. This indicates that pearl hydrolysate may have a positive effect on protecting the function of vascular endothelial cells (Cen et al., 2018).

#### 4.3.3 Anti-Haemolytic Effect

2,2′-Azobis (2-amidinopropane) dihydrochloride (AAPH) is a water-soluble free radical generator that induces hemolysis in cells. In human erythrocyte culture *in vitro*, pretreatment of erythrocytes with pearl powder can resist AAPH-induced oxidative hemolysis, manifesting as a significant reduction in AAPH-induced hemolysis (Yang et al., 2017). This suggests that pearls can be used as a new therapeutic medicine for hemolytic diseases.

### 4.4 Protective and Repair Effects on the Skin and Other Tissues

#### 4.4.1 Anti-Oxidative Effect

During the oxidation process, the presence of (1,1-diphenyl-2-picrylhydrazyl) radical (DPPH·), 2,2′-azinobis (3-ethylbenzothiazoline-6-sulfonic acid) radical (ABTS·), hydroxyl radical (OH·), and superoxide anion radical (O_2_^−^·) would aggravate the oxidation reaction; meanwhile, SOD and glutathione peroxidase (GSH-Px) would inhibit the oxidative process (Hu et al., 1994; Yang A. Q. et al., 2015; Pu et al., 2017; Pu W. et al., 2018; Liu et al., 2020; Liu et al., 2020).

Pearls have been proven to be an excellent antioxidant. In terms of scavenging free radicals, seawater pearl hydrolysate and freshwater pearl hydrolysate showed strong scavenging ability to DPPH and ABTS·, whereas their scavenging effect on OH· and O_2_^−^· was relatively weak. However, the scavenging rate of all free radicals increased with the increase in volume fraction of pearl hydrolysate, and the concentration of pearl hydrolysate used was much lower than that of ascorbic acid at an equivalent scavenging rate (Pu et al., 2017; Pu W. et al., 2018). In addition, pearl powder, protein extracts in pearl powder, and non-protein extracts in pearl powder showed a scavenging effect on DPPH· and O_2_^−^·, and the scavenging ability of protein extracts was stronger (Chiu et al., 2018), indicating that the antioxidant capacity of pearls may depend on its protein composition. However, a study showed that the preliminary purification protein sample of pearls (macroporous resin) did not exhibit obvious scavenging ability for DPPH· and O_2_^−^· but had strong scavenging ability for OH·(Liao, 2019). The reason for the different results above may be related to the extraction method of pearls. Moreover, the preliminary purified protein sample may be only a part of the total protein, and the effective protein has not been presented.

In terms of the effect on oxidative enzymes, pearl extracts have a SOD-like effect, which can replace SOD to scavenge free radicals (Yang YL. et al., 2015). Pearl hydrolysate can improve SOD activity and reduce lipid peroxide generation in medium-aged rats (Hu et al., 1994). *In vitro* experiments also showed that pearl hydrolysate can enhance GSH-Px activity in human lens epithelial cells and microvascular endothelial cells, reduce glutathione, decrease malondialdehyde, scavenge active free radicals, and protect cells from H_2_O_2_-induced oxidative damage (Zheng and Mao, 2004; Liu et al., 2020a; Liu et al., 2020b). Antioxidation also has a role in prolonging the life span. Huang and Pan (2000) and Ma and Xiao (2007) showed that timely administration of pearl powder at a young age of *Drosophila* could improve its vitality. It can be seen from the above that the action of enzymes can affect the scavenging of free radicals, indicating that the oxidation process is a chain reaction. Thus, further research is needed to better reveal the antioxidant mechanism of pearl powder.

#### 4.4.2 Strengthen Immunity

The functions of T and B lymphocytes and mononuclear phagocytes can reflect the immune function *in vivo*. After taking pearl powder, the proportion of T lymphocytes in peripheral blood and the ratio of spleen antibody formation improved, and the phagocytosis of neutrophils in peripheral blood was enhanced (Wang et al., 1994). In addition, pearl powder can also improve the level of serum hemolysin and the activity of natural killer cells in mice (Qian and Zhu, 2003). Similarly, the better-absorbed pearl hydrolysate can also enhance the cellular immunity and humoral immunity of immunocompromised mice. The hydrolyzed Nanzhu tablet can improve the carbon clearance index K of the monocyte macrophage system and the number of T lymphocytes in peripheral blood and promote the production of serum hemolysin (Lan et al., 2017). Hydrolysis of the seawater pearl tablet can reduce the proportion of spleen CD3^+^/CD4^+^ T lymphocytes and plays an immunomodulatory role (Chen et al., 2020). To sum up, pearl powder and pearl hydrolysate have good immunity-enhancing functions, but their respective advantages are unclear. Thus, comparative research needs to be carried out.

A new form of pearls has also been developed as a regulator of Th1 and Th2 immune cells. Cell and molecular level studies confirmed that pearl in ash form increased Toll-like receptor-2 (TLR-2) and specific lymphocytes on murine peritoneal macrophages and improved total immunoglobulin G, immunoglobulin G1, immunoglobulin G2a, and immunoglobulin G2b levels (which were still higher than the control group at 60 days after immunization). In particular, the dose of 50 μg/kg exerted the most significant effect, indicating that pearl in ash form can not only enhance the immune response of the body, but also have a long-term effect. This effect may be mediated by the activation of the TLR-2 signaling pathway to induce the interferon-beta- (TRIF-) dependent pathway, leading to the activation of T cells, which in turn promotes an effective immune response (Elahi et al., 2014). The pearl in ash form is an aggregate of mineral compounds, suggesting that inorganic elements may be its effective constituents.

In addition, pearl pulvis, the compound preparation of pearls, has the function of enhancing the body’s resistance. This effect is achieved by promoting the production of serum hemolysin and significantly improving the phagocytic function of macrophages and the proliferation function of spleen T lymphocytes in immunocompromised mice (Liu et al., 2003).

#### 4.4.3 Promote Wound Healing

The chemical constituents in pearls have a significant role in promoting wound healing. For example, as the main component, calcium can alleviate the permeability of capillaries and reduce exudates, which is conducive to the growth of fresh granulation; potassium and sodium are anti-inflammatory and antiseptic; and zinc can accelerate tissue repair (Mo et al., 2015).

Studies have shown that pearl hydrolysate (Zheng and Mao, 2004), compound preparation Zhushen pulvis (Lin et al., 1996), Zhuhuang ointments (Liu et al., 1995), Zhenzhu Shaoshang ointments (Huang et al., 1997), and Zhenzhu pulvis (Kan et al., 2015) have excellent anti-inflammatory and anti-scalding effects, mainly manifested by the inhibition of various swelling, attenuation of capillary permeability, and promotion of wound healing in second-/third-degree burns. During this process, the level of basic fibroblast growth factor was significantly increased, and the value-added rate of skin cells increased. *In vitro* experiments also showed that pearl extracts and poly(γ-glutamic acid) combined to form a hydrogel could treat inflammation in human keratinocyte cells (HaCaT cells) caused by ultraviolet radiation B irradiation (Yang YL. et al., 2015). In addition, pearls have a good repair effect on skin or mucosal ulcers. In a rat model of gastric mucosal injury, the ulcer index was significantly reduced after treatment with nacre powder; however, the efficacy of ultrafine nacre powder was better (Xia et al., 2014). In short, the role of pearls in promoting wound healing is closely related to their anti-inflammatory, anti-scalding, and anti-ulcer effects.

#### 4.4.4 Whitening Skin Care

Pearls have been used as a whitening skin care product as early as 4,000 years ago; for example, ancient Egyptian women used pearls with milk to wipe their bodies, and China also used pearls as an important medicine for cosmetology in the traditional medical book *Ming Yi Bie Lu* during the San Guo (AD 220–280) (Zhang et al., 2002).

Many scholars have studied the skin-whitening efficacy of pearls by using modern pharmacological research means. After the extraction of pearls with different solvents, they all had a certain moisture absorption rate and moisturizing rate, but some were more hygroscopic and adept at moisturizing, suggesting that their combination may lead to better skin care outcomes (Yang et al., 2016b). Whilst the occurrence of the whitening effect is inseparable from the production of melanin and the activity of tyrosinase, studies have found that pearl extracts (Yang et al., 2016b; Shen, 2017; Yang et al., 2018a; Liao, 2019) and hydrolyzed pearl (Pu and Tong, 2015; Deng et al., 2017) can reduce the activity of tyrosinase in B16 melanoma cells, thereby inhibiting melanin production. In this process, the mRNA expression of the tyrosinase gene, tyrosinase-related protein 1, and microphthalmia-associated transcription factor was suppressed. Moreover, a dipeptide compound with the molecular formula C_11_H_12_N_2_O_2_ was identified as one of the pearls’ active constituents (Liao, 2019).

#### 4.4.5 Protect Eyes and Repair Eyesight

Pearls also have eye protection and vision restoration effects. The pearls were ground into powder and applied to injured rabbit corneas. The pathological conditions of rabbit corneas were significantly improved and almost indistinguishable from those of normal rabbits, and their corneal pannus changes were observed at 3 months after discontinuation of the drug. The results showed that the corneal pannus thickness was significantly reduced to within 2 mm, similar to the effect of clinically used Zhenzhu Mingmu eye drops, which reduce or remove scars (Lu et al., 1990). The effect of nacre powder is also not weaker than that of pearl powder. After using nacre powder hydrolysate to treat rabbits with eyeball microcirculation disorder model, it was found to increase the number of capillary crossings of the ocular conjunctiva and effectively improve the microcirculation of the eyeball (Gao et al., 2000).

Fufang Zhenzhu hydrolysate and Zhenzhu pills also have the effect of protecting the eyes and restoring vision. After treating the deprivation myopia model in chickens with Fufang Zhenzhu hydrolysate, the expansion of the outer diameter, inner diameter, and equatorial radius of the eyeballs was inhibited, indicating that the medicine has a repairing effect on vision (Chen et al., 2001). The eye protection effect of Zhenzhu pills is mainly reflected in their ability to treat retinal ischemia-reperfusion injury in rabbits (Meng et al., 2007).

#### 4.4.6 Anti-Neoplastic Effect

Chen and Yuan (1990) extracted and isolated porphyrin compounds from pearl powder, and the results showed that porphyrin compounds can inhibit S_180_ sarcoma and Lewis lung cancer tumors, in which the inhibition rate of S_180_ sarcoma reached 34.8%, and the inhibition rate against Lewis lung cancer tumors was 13.89%. In addition, porphyrin compounds can prolong the survival time of P_388_/J lymphocytic leukemia mice and reduce the spleen weight of animals, indicating that it has a certain inhibitory effect on P_388_/J lymphocytic leukemia, and this inhibitory effect is mainly achieved through killing P_388_/J_3_ cells. However, more experiments are needed to confirm the anti-neoplastic effect (Chen and Yuan, 1990).

#### 4.4.7 Anti-Apoptotic Effect

Studies have shown that pearl extracts can inhibit H_2_O_2_-mediated apoptosis of human skin fibroblasts and ultraviolet-mediated apoptosis of human keratinocytes to a certain extent (Yang et al., 2018b; Wang et al., 2018). In addition, the nacre water-soluble organic matrix and the preparation of Zhenzhu pills also showed anti-apoptotic effects on osteoblasts and retinal neuron cells, respectively. After using them, the survival rate of osteoblasts increased significantly, and the number of apoptotic retinal neuron cells decreased significantly. In this process, B cell lymphoma-2 (Bcl-2) gene expression was promoted (Mouriès et al., 2002; Meng et al., 2007).

#### 4.4.8 Antibacterial Effect

Pearl extracts (240 mg/ml) have a strong inhibitory effect on *Staphylococcus aureus*, and its antibacterial circle reached 17.861 ± 0.948 mm. In addition, the antibacterial activity is almost unaffected regardless of the influence of different temperatures, strong acid and alkali, ultraviolet radiation, metal ions, or pancreatic pepsin (Liu et al., 2020). Another compound preparation containing pearls, Zhenzhu Shaoshang ointments, also showed an inhibitory effect on *Pseudomonas aeruginosa* (Hu et al., 1998).

## 5 Toxicology of Pearls

As a valuable and common Chinese medicinal material with a specific good curative effect, pearls have been used since ancient times. With the popularization of its application, its toxicity has attracted more and more attention. Ancient Chinese medicinal books have recorded the safety of pearls. For example, Kai Bao Ben Cao (AD 974) said, “Non-toxic.” Shao Xing Ben Cao (AD 1159) also indicated, “Slightly cold, non-toxic (Medicine, 1999).” It can be seen that pearls as medicine have been proven to be safe since ancient times.

In modern pharmacology, much related research on the toxicity of pearls has been conducted. In terms of cytotoxicity, Zhang (2019) and Mao et al. (2018) showed that cells exposed to nano-pearl powder or nano-pearl powder extracts did not exhibit large area abscission or obvious cell morphological changes caused by cell damage. However, when the concentration was high (500 μg/ml or 100% extracts), the cell proliferation rate was relatively reduced, and the extracellular matrix was poorly extended and had certain cytotoxicity (Mao et al., 2018; Zheng et al., 2019). Although the toxicity of high and low concentrations is different, the rating is grade 1, which meets the application standard of biomaterials in China. In terms of acute toxicity, the histopathology of the abdominal cavity and internal organs was observed after the intraperitoneal administration of nano-pearl powder extracts to rats, and there were no abnormalities (Mao et al., 2018). Water-soluble pearl powder, pearl powder, and dyed black pearls were administered to mice at the maximum dose (10, 15, and 10 g/kg, respectively). Similarly, no obvious poisoning symptoms were found, and no animals died within 14 days (Wu, 2009; Shen et al., 2013; Deng et al., 2014). In terms of skin irritation, black pearls were studied and showed no primary irritation to rabbit skin (Deng et al., 2014). In terms of intradermal stimulation, the nano-pearl powder extracts were injected into the back skin of rabbits. After 4 h, the skin mounds disappeared at each injection site, and no skin erythema and edema were observed at 24, 48, and 72 h (Mao et al., 2018). In terms of genotoxicity, neither water-soluble pearl powder nor pearl powder showed mutagenic effects on mice sperm, indicating that they were not genotoxic (Wu and Zhu, 2009; Shen et al., 2013). In terms of long-term toxicity, the general condition, body weight, food utilization rate, hematology, blood biochemistry, visceral body ratio, and histopathology of rats after taking water-soluble pearl powder and pearl powder were detected through a 30-day feeding experiment, and the results were normal (Wu and Zhu, 2009; Shen et al., 2013).

In summary, all forms of pearls are non-toxic and non-irritating in terms of cytotoxicity, acute toxicity, skin irritation, intradermal irritation, genotoxicity, and long-term toxicity, indicating the safety of pearls for internal and external use.

## 6 Clinical Applications of Pearls

See Table 7 for details.

**TABLE 7:** Results of clinical trials of pearls.

Diseases	The state, preparations, or combination medication of pearls	Experimental subjects	Research design	Grouping and number of people		Treatment ,ethod		Course of treatment	Results	References
				Treatment group	Control group	Treatment group	Control group			
Convulsions, epilepsy	Preparations: Angong Niuhuang pills	70 children (46 males, 24 females)	Randomized controlled experiment	36	34	Routine care + chloral hydrate or diazepam + Angong Niuhuang pills (once a day, 1/6 pill to 1/2 pill each time), oral	Routine care + chloral hydrate or diazepam (1 mg/kg/d, divided three times), oral	5 d	The total effective rate in the treatment group was 86.11%, with three cases of recurrence; the total effective rate in the control group was 79.41%, with 11 cases of recurrence; and the convulsion time in the treatment group was shorter than that in the control group	Zhong et al. (2010)
		25 children (14 males, 11 females)	Randomized controlled experiment	25 (14 males, 11 females)	——	Routine care + chloral hydrate or diazepam + Angong Niuhuang pills (once a day, 1/6 pill to 1/2 pill each time), oral	Routine care + chloral hydrate or diazepam (1 mg/kg/d, divided three times), oral	2∼3 d	Angong Niuhuang pills is less and shorter than diazepam in the frequency and duration of seizures	Zhu et al. (2011)
	Preparations: Qishiwei Zhenzhu pills	80 patients (43 males, 37 females)	Randomized controlled experiment	40 (21 males, 19 females)	40 (22 males, 18 females)	Qishiwei Zhenzhu pills (1 g every 7 days, 1 g per day for the seriously ill), oral	Oryzanol (three times a day, 10 mg each time), oral	14 d	The total effective rate of the treatment group was 87.5%, and that of the control group was 77.5%, the therapeutic effect of the treatment group is better than that of the control group	Sun and Qiu (2012)
Palpitations	Pearl powder + Amber powder	42 patients (18 males, 24 females)	Randomized experiment	42 (18 males, 24 females)	——	Basic formula + pearl powder (0.3 g) + amber powder (1 g), two times a day, oral	——	7 d	19 cases were cured, 20 cases were improved, and three cases were ineffective. Suggesting a significant efficacy	Yu (2013)
	Preparations: Zhenzhu Anshen syrups	80 patients (16 males, 64 females)	Randomized controlled experiment	40 (8 males, 32 females)	40 (8 males, 32 females)	Zhenzhu Anshen syrup (three times a day, 20 ml each time, plus one time before going to bed), oral	Diazepam tablets (one time per night, two tablets before bedtime), oral	28 d	The total effective rate in the treatment group was 92.5%, which was higher than 82.5% in the control group	Wu (2009)
Eye diseases	Preparations: Zhenzhu Jingming tablets	877 patients (574 males, 303 females)	Randomized controlled experiment	451 (293 males, 158 females)	426 (281 males, 145 females)	Zhenzhu Jingming tablets (three times a day, four tablets each time), oral	Zhangyanming tablets (three times a day, four tablets each time), oral; Baineiting eye drops (four times a day, one drop each time), eye drops	3 m	The total effective rate in the treatment group was 86.97%, which was significantly higher than 67.91% in the control group. Zhenzhu Jingming tablets are more effective in preventing and treating early senile cataract	Li and Gao (2006)
	Preparations: Zhenzhu Mingmu eye drops	76 patients (28 males, 48 females)	Randomized controlled experiment	38 (13 males, 25 females)	38 cases (15 males, 23 females)	Zhenzhu Mingmu eye drops (3–5 times a day, 1 to 2 drops each time), eye drops	0.25% chloramphenicol eye drops (3–5 times a day, 1 to 2 drops each time), eye drops	4w	The effective rate of the treatment group was 89.47%, and the markedly effective rate was 34.21%; the effective rate of the control group was 36.84%, and the markedly effective rate was 10.53%, and the improvement of ocular symptoms in the treatment group was better than that in the control group	Niu and Xu (2005)
	Preparations: Zhenzhu Tuiyi pulvis	24 patients (6 males, 18 females)	Randomized experiment	24 (6 males, 18 females)	——	Evenly place the Zhenzhu Tuiyi pulvis on the inner edge of the upper eyelid, close your eyes for 10–15 min, 3–5 times a day	——	——	13 cases were cured, accounting for 54%; 9 cases were effective, accounting for 38%; 2 cases were ineffective, accounting for 8%; and the total effective rate was 92%. Predicting a significant effect	Wang (1996)
Oral ulcer	Pearl powder + metronidazole + vitamin B2 etc.	200 patients (109 males, 91 females)	Randomized controlled experiment	100 (55 males, 45 females)	100 (54 males, 46 females)	Apply metronidazole + vitamin B2 + pearl powder (three times a day) to the affected area	Watermelon frost spray (three times a day)	7 d	The total effective rate in the treatment group was 97%, higher than 82% in the control group, the recurrence rate was significantly lower than that in the control group, and the pain relief time and ulcer healing time were significantly shorter than those in the control group. During the period, the levels of CD4^+^ and CD4+/CD8+ increased significantly in the treatment group	Zeng and Liu (2017)
	Pearl powder + vitamin B2	100 patients (46 males, 54 females)	Randomized controlled experiment	50 (24 males, 26 females)	50 (22 males, 28 females)	Pearl powder capsules + vitamin B2 (two times a day, 0.6 g each time), oral	Vitamin B2 (three times a day, 5 mg each time), oral	24 w	The prolongation of the intermittent period of recurrent ulcers and the reduction of the number of ulcers in the treatment group were significantly better than those in the control group, and the total effective rate in the treatment group reached 100%, compared with 98% in the control group	Shao et al. (2018)
	Pearl powder + lidocaine	102 patients (46 males, 56 females)	Randomized controlled experiment	51 (24 males, 27 females)	51 (22 males, 29 females)	Spread pearl powder + lidocaine (three–five times a day) on the affected area	Guilin watermelon frost (three–five times a day) spray on the affected area	3 d	The cure rate of the treatment group (88.24%) was significantly higher than that of the control group (64.71%), and the pain relief status of the patients in the treatment group was excellent	Fu (2014)
Duodenal ulcer	Pearl powder + ranitidine	112 patients (81 males, 31 females)	Randomized controlled experiment	54 (40 males, 14 females)	58 (41 males, 17 females)	Pearl powder (0.3 g) + ranitidine (0.15 g) (once half an hour before meals of the morning and evening), oral	Ranitidine (0.15 g each time, once half an hour before breakfast and dinner), oral	4 w	The healing rate of the treatment group was 95.6%, the abdominal pain disappearance rate was 87.3%, and the control group was 74.9% and 41.5%, respectively. The difference between the two groups was significant	Zuo (1995)
Stress ulcer bleeding	Pearl powder + Yunnan Baiyao	51 patients (27 males, 24 females)	Randomized controlled experiment	26 (14 males, 12 females)	25 (13 males, 12 females)	Yunnan Baiyao capsules (0.5 g) + pearl powder capsules (0.6 g), gavage	Omeprazole capsules (20 mg), gavage	2 w	The total effective rates of the observation group and the control group were 96.15% and 96.00%, respectively. The effect of Yunnan Baiyao combined with pearl powder and omeprazole is equivalent	Xie et al. (2015)
Pressure sores	Pearl powder	65 patients (38 males, 27 females)	Randomized controlled experiment	35	30	Regular care + pearl powder (two times a day, 1 mm each time), apply	Routine care + ofloxacin (two times a day, 1 mm each time), apply	10 d	The cure rate of the treatment group was 82.86%, and the cure rate of the control group was 36.67%. The healing time of pressure ulcers and local nursing time in the treatment group were shorter than those in the control group	Xie (2013)
		21 patients (9 males, 12 females)	Randomized experiment	21 (9 males, 12 females)	——	Routine care + sterilized pearl powder (dressing once a day), apply	——	——	The scab formed after 1 day of the dressing was changed, and the scab fell off and healed after 2 days. Pearl powder, especially sterilized pearl powder, is effective in treating bedsores	Zhang et al. (2013)
	Pearl powder + Baiduobang ointments + sesame oil	80 patients (48 males, 32 females)	Randomized experiment	80 (48 males, 32 females)	——	Baiduobang ointments + pearl powder + sesame oil (dressing once a day, 1 mm each time)	——	15 d	The total effective rate was 97.5%, and the cure rate was 81.3%	Huang and Qiu (2013)
	Pearl powder + norfloxacin	96 patients (55 males, 41 females)	Randomized controlled experiment	48 (28 males, 20 females)	48 (27 males, 21 females)	Routine care + pearl powder + norfloxacin (two times a day, 60s each time), external application	Routine care (two times a day)	10 d	The total effective rates of the treatment group and the control group were 93.73 and 81.25%, respectively. The effect of the treatment group was significantly better than that of the control group, and the time to good outcome was less than in the control group	Wang and Chen (2015)
	Pearl powder + compound aescin gel	80 patients (47 males, 33 females)	Randomized controlled experiment	40 (25 males, 15 females)	40 (22 males, 18 females)	Compound aescin sodium gel + pearl powder (used alternately, 3–4 times a day), apply	Saifurun (3–4 times a day), apply	——	The total effective rate of the treatment group was 97.5%, and the total effective rate of the control group was 62.5%. The treatment group had a significant curative effect	Tang and Wang (2016)
	Pearl powder + rivanol	10 patients (7 males, 3 females)	Randomized experiment	10 (7 males, 3 females)	——	Routine care + rivanol + pearl powder (change dressing two–three times a day), apply	——	2∼3 w	After 3–5 days, the patient’s sores were dry, and after 2–3 weeks, the patient’s pressure sores were healed. Rivanol dressing change combined with external application of pearl powder is effective in the treatment of pressure ulcer above stage of Ⅱ	Li and Fu (2012)
	Pearl powder + erythromycin ointments	70 patients (43 males, 27 females)	Randomized controlled experiment	35 (18 males, 17 females)	35 (20 males, 15 females)	Routine care + pearl powder + erythromycin ointment (two times a day), apply	Routine care	4 w	The total effective rate in the treatment group was 97.14%, which was higher than 85.71% in the control group	Fang and Hung (2013)
	Pearl powder + anputie	60 patients (36 males, 24 females)	Randomized controlled experiment	30 (19 males, 11 females)	29 (17 males, 12 females)	Routine care + pearl powder + anputie (dressing once every 2 days), external application	Routine care + anputie (dress change once every 2 days), external application	1∼2 w	The total effective rate in the treatment group was 96.7%, which was better than 79.3% in the control group, and the healing time and markedly effective time in the treatment group were shorter than those in the control group	Lai (2013)
Dermatitis	Pearl powder + clotrimazole	91 patients (53 males, 38 females)	Randomized controlled experiment	46	45	Clotrimazole + pearl powder (three times a day), apply	Dakening ointments (three times a day), apply	7 d	The total effective rate in the treatment group was 97.83%, and the significant effective rate was 67.39%, which was better than 93.33% and 46.67% in the control group, and the effective time in the treatment group was shorter than that in the control group	Xu et al. (2014a)
	Pearl powder + zinc oxide ointments	60 neonatal patients (36 males, 24 females)	Randomized controlled experiment	30	30	Routine care + pearl powder + zinc oxide ointments, apply	Regular care + zinc oxide ointments, apply	3 d	The cure rate of the treatment group was 56.67%, and the total effective rate was 100%; the cure rate of the control group was 30.00%, and the total effective rate was 83.33%	He (2019)
	Pearl powder + tin powder + sesame oil	78 infantile patients (38 males, 40 females)	Randomized controlled experiment	40 (21 males, 19 females)	38 (17 males, 21 females)	Regular care + pearl powder + tin powder + sesame oil (three times a day), apply	Routine care + erythromycin ointments, apply	2–10 d	The total effective rate of the treatment group was 95.0%, which was significantly higher than 65.8% of the control group	Dai (2015)
	Pearl powder + zinc oxide	90 patients (30 each in pearl powder group, zinc oxide group and combination medication group)	Randomized controlled experiment	30	60	Regular care + pearl powder + zinc oxide (three times a day, once 0.5 mm), apply	Routine care + pearl powder (three times a day, once 0.5 mm), apply	3, 6, 9 d	After 9 days of treatment, the total effective rate of the pearl powder group was 90.0%, that of the zinc oxide group was 93.3%, and the combination group was 100.0%. The combination group was significantly better than the pearl powder group and the zinc oxide group	(Chen, 2017; Chen et al., 2019b)
							Routine care + zinc oxide (three times a day, once 0.5 mm), apply			
Chloasma	Preparations: Luhui Zhenzhu capsules	66 patients (female)	Randomized controlled experiment	30	30	Luhui Zhenzhu capsules (two times a day, two capsules each time, orally; one time every 3 days, six capsules each time, external use) + vitamin C + vitamin E	Vitamin C + vitamin E	8 w	The effective rate was 94.83% in the treatment group and 70.00% in the control group, which was significantly better than the control group. The recovery of testosterone, estradiol, progesterone, follicle-stimulating hormone, luteinizing hormone, and prolactin levels was more pronounced in the treatment group during this process	Jing (2015)
Eczema	Pearl powder + compound glycyrrhizin	60 patients (33 males, 27 females)	Randomized controlled experiment	30 (16 males, 14 females)	30 (17 males, 13 females)	Compound glycyrrhizic acid tablets (three times a day, two tablets each time) + pearl powder capsules (two times a day, two tablets each time), oral	Compound glycyrrhizic acid tablets (three times a day, two tablets each time), oral	4 w	The levels of IL-2, IL-6, and CRP in the two groups decreased to a greater extent than those in the treatment group for each index; the total effective rate in the treatment group was 100%, which was greater than 86.67% in the control group	Pu et al. (2018b)
Burns and scalds	Preparations: Zhenzhu pulvis	150 patients (92 males, 58 females)	Randomized controlled experiment	75 (44 males, 31 females)	75 cases (48 males, 27 females)	Routine care + erythromycin ointments + pearl powder (once a day or every other day), apply	Routine care (once a day or every other day)	——	The addition of Zhenzhu pulvis can significantly reduce the pain of patients, reduce the number of dressing changes, shorten the healing time, and effectively promote wound healing	Long (2014)
Skin ulcer	Pearl powder + Kangfuxin solution	16 patients (10 males, 6 females)	Randomized experiment	16 (10 males, 6 females)	——	Basic care + Kangfuxin solution + sesame oil + pearl powder, stir evenly and apply it to the sore surface (change the dressing once or twice a day)	——	30 d	Eight cases were cured, seven cases were improved, and one case was effective. The total effective rate is 100%	Jiang (2013)
	Preparations: Zhenzhu pulvis	100 patients (74 males, 26 females)	Randomized experiment	100 (74 males, 26 females)	——	Spread the Zhenzhu pulvis on the wound surface (usually change the dressing once every 2–3 days)	——	——	Zhenzhu pulvis has a good therapeutic effect on chronic skin ulcer	Zhu (2007)
Acne	Preparations: Meijie Shuirong Zhenzhufen capsules	60 patients (17 males, 43 females)	Randomized controlled experiment	30 (9 males, 21 females)	30 (8 males, 22 females)	Enzymatic hydrolyzed water-soluble pearl powder capsules (two times a day, two capsules each time), oral	Minocycline (twice a day, 50 mg each time), oral	3 m	The cure rate was 73.3% in the treatment group and 70% in the control group. The improvement in the treatment group was more obvious	Xiao et al. (2015)
Cervical erosion	Pearl powder	94 patients (female)	Randomized controlled experiment	64	30	Spray pearl powder on the affected area (once a day, 0.1–0.2 g each time)	Chlorhexidine (once a day)	10 d	In the observation group, the apparent efficiency reached 80%, the improvement rate was 17%, and the total effective rate was 97%, which was superior to the 30 cases of in the control group (chlorhexidine)	Tang et al. (1992)
Infection of the perineal side incision	Pearl powder	47 parturients	Randomized controlled experiment	25	22	Pearl powder (twice a day), apply	0.1% rivanol (dressing once a day), for external application	2 w	The recovery rate of perineal incision in the treatment group was 80%, which was significantly higher than 27.27% in the control group, and the pain relief in the treatment group was better	Zhao and Yu (2014)
Perianal infections	Pearl powder + Saimeian pulvis	60 patients (34 males, 26 females)	Randomized controlled experiment	30 (16 males, 14 females)	30 (18 males, 12 females)	Saimeian pulvis + pearl powder, apply	Silver sulfadiazine cream, apply	——	In the treatment group, 18 cases were markedly effective, 10 cases were effective, and 2 cases were ineffective; in the control group, 6 cases were markedly effective, 19 cases were effective, and 5 cases were ineffective. The effect of the treatment group was better than that of the control group	Chen (2015)

Note: in the table, “d” means days, “w” means weeks, and “m” means months.

### 6.1 Convulsions and Epilepsy

Compound preparations containing pearls are commonly used to treat convulsions and epilepsy. For example, Angong Niuhuang pills are used for opening the orifices and awaking the spirit. Zhong et al. (2010) divided 70 children with febrile convulsion into the treatment and control groups. The treatment group was treated with Angong Niuhuang pills, with a total effective rate of 86.11%, which was better than the control group with conventional treatment, and the time to stop convulsions and recurrence rate were significantly shortened and reduced. In another study, after 25 children with febrile convulsion were treated with Angong Niuhuang pills, the number of seizures and duration of convulsion were reduced and shortened (Zhu et al., 2011).

### 6.2 Palpitations and Insomnia

In 42 patients with phlegm-stasis block and heart and spleen deficiency accompanied by palpitations, Yu (2013) added pearl powder and amber powder to the basic prescription, which resulted in a satisfactory curative effect: 19 cases were cured, 20 cases improved, and 3 cases were invalid (Yu, 2013). Sun and Qiu (2012) used the compound preparation of pearls, Qishiwei Zhenzhu pills, to treat 40 of 80 patients with insomnia, and the results showed that the total effective rate was 87.5%, which was significantly higher than that of the control group treated with oryzanol, indicating that Qishiwei Zhenzhu pills have a better therapeutic effect on insomnia (Sun and Qiu, 2012). Another compound preparation of pearls, Zhenzhu Anshen Syrup, was also used to treat 40 patients with insomnia and showed a total effective rate of 92.5%, which was higher than that of the diazepam-treated control group (82.5%) (Wu, 2009).

### 6.3 Eye Diseases

The compound preparation Zhenzhu Jingming tablets are used to prevent and treat the early senile cataract. In a clinical study, 877 patients with early senile cataract were divided into the treatment and control groups. The treatment group was treated with Zhuzhu Jingming tablets, whereas the control group was treated with Zhangyanming tablets and Baineiting eye drops. The results showed that the total effective rate was 86.97% in the treatment group and 67.91% in the control group. Zhenzhu Jingming tablets showed better efficacy (Li and Gao, 2006). In addition, amongst 76 patients with chronic conjunctivitis, the total effective rate of 38 patients treated with Zhuzhu Mingmu eye drops was significantly higher than that of 0.25% chloramphenicol (Niu and Xu, 2005). In another study, Zhenzhu Tuiyi pulvis was used to treat 24 patients with coiled filamentous keratitis, and the total effective rate reached 92% (Wang, 1996).

### 6.4 Ulcer Diseases

Pearl powder alone or in combination with other drugs can be used to treat various ulcer diseases, such as oral, duodenal, and stress ulcer bleeding.

In oral ulcers, Zeng and Liu (2017) selected 200 patients with recurrent oral ulcers (Zeng and Liu (2017). After 7 days, the total effective rate of the treatment group (metronidazole + vitamin B_2_ + pearl powder) was 97%, which was significantly higher than that (82%) of the control group (watermelon frost spray). Moreover, the recurrence rate, pain sensation elimination time, and ulcer healing time were significantly lower than those in the control group. During this period, the levels of CD4^+^ and CD4^+^/CD8^+^ of the two groups in the peripheral blood were increased, and the increase was more significant in the treatment group. Shao et al. (2018) also used pearl powder combined with vitamin B_2_ to treat 50 patients with recurrent oral ulcers, and the total effective rate reached 100%, which was higher than that of vitamin B_2_ alone. Moreover, the number and the interval of ulcers were significantly reduced and prolonged, indicating that the addition of pearl powder produced faster and better recovery of oral ulcers (Shao et al., 2018). In addition, pearl powder combined with lidocaine was used to treat 51 patients with oral ulcers of grades II–III. The results showed that the cure rate reached 88.24%, which was significantly better than that of the control group treated with watermelon frost, and the pain was also significantly relieved (Fu, 2014).

In duodenal ulcers, the combination of pearl powder and ranitidine has a significant effect. A total of 112 duodenal ulcer patients were taken as the research object. After 4 weeks, the healing rate and abdominal pain disappearance rate of the treatment group (pearl powder + ranitidine) were 95.6% and 100%, respectively, which were significantly higher than those (74.9% and 83%, respectively) of the control group (ranitidine), further proving that the presence of pearl powder made the treatment of duodenal ulcer more prominent (Zuo, 1995).

In stress ulcer bleeding, Yunnan Baiyao combined with pearl powder can effectively treat stress ulcer bleeding caused by senile dementia. A total of 51 patients with senile dementia complicated with stress ulcer bleeding were randomly divided into the treatment group and control group. The treatment group was treated with Yunnan Baiyao combined with pearl powder, whereas the control group was treated with omeprazole. The results showed that the levels of the two groups were equivalent, and both could effectively stop bleeding and repair ulcers (Xie et al., 2015).

### 6.5 Skin Diseases or Skin Injuries

Common skin diseases or skin injuries include pressure ulcers, dermatitis, chloasma, eczema, burns, skin ulcers, and acne, each of which can be treated with pearl powder as a primary or adjunct treatment.

Pressure ulcers, also known as bedsores, are a major cause of common skin problems. Xie (2013) used pearl powder to treat 35 patients with pressure ulcers, and the results showed that the healing time and local nursing time of pearl powder were significantly lower than those of ofloxacin gel. The cure rate of pearl powder reached 82.86%, which was significantly higher than that of 36.67% of the control group. In another study, pearl powder combined with Baiduobang ointments and sesame oil was used to treat 80 patients with pressure ulcers of stages III–IV. The total effective rate reached 97.5%, and the recovery rate was 81.3% (Huang and Qiu, 2013). The total effective rate also reached 93.75% in 48 pressure ulcer patients treated with the combination of pearl powder and norfloxacin, and the time to improvement was significantly shorter than that in the control group (Wang and Chen, 2015). After treatment with pearl powder and compound aescin gel in 40 patients with pressure ulcers, the average healing time and total effective rate were both shorter and higher than those of saifurun (Tang and Wang, 2016). The combination of pearl powder and rivanol also showed a good effect in the treatment of 10 patients with pressure ulcers (Li and Fu, 2012). In another study, pearl powder and erythromycin ointments were added on the basis of routine nursing to treat 30 patients with decubitus ulcers, and the results showed that the total effective rate was 97.14%, which was higher than that of routine nursing (Fang and Huang, 2013). Similarly, when 30 decubitus ulcer patients were treated with pearl powder combined with anputie, the healing time and marked effect time were significantly shorter than those of the control group, and the total effective rate reached 96.7% and 100% after 1 and 2 weeks, respectively (Lai, 2013). The above examples all show that pearl powder has a significant effect in treating pressure ulcers.

For the treatment of dermatitis, in a clinical study, 91 patients with mycotic dermatitis were randomly divided into two groups: one group was treated with clotrimazole and pearl powder and the other with Dakening ointments. A significant effective rate of 67.39% was found in the group treated with clotrimazole and pearl powder, which was significantly superior to that of 46.67% in the group treated with Dakening ointments. Moreover, the duration of treatment was shorter in the former (Xu X. L. et al., 2014). In another study, 30 neonates with severe diaper dermatitis treated with zinc oxide ointments combined with pearl powder showed a total effective rate of 100%, which was significantly higher than the total effective rate of zinc oxide ointments alone, suggesting that pearl powder played an important role during this period (He, 2019). In another clinical experiment, pearl powder, tin powder, and sesame oil were used to treat 40 infants with neonatal diaper dermatitis. The total effective rate was 95%, and the total effective rate of the buttock cream was only 65.8%, showing a significant difference (Dai, 2015). Pearl powder combined with zinc oxide can also be used to treat incontinence dermatitis, and in 90 patients, the total effective rate of the combined medication was 100%, which was higher than the total effective rate of pearl powder or zinc oxide alone, indicating that combined medication can treat incontinence dermatitis faster and more effectively (Chen, 2017; Chen X. et al., 2019).

Regarding chloasma, Jing (2015) conducted a clinical study on 60 patients with chloasma, and the results showed that the total effective rate of the treatment group (Luhui Zhenzhu capsules + vitamin C + vitamin E) was 87%, which was higher than that of 70% in the control group (vitamin C + vitamin E) (Jing, 2015). During this process, the levels of estrogen such as estradione, progesterone, luteinising hormone, follicle-generating hormone, testosterone, and prolactin were decreased in the two groups, but the decrease was more obvious in the treatment group, in which pearl powder played a great promoting role. In addition, Wei and Feng (2014) and Zhang X. et al. (2014) statistically analyzed the outcomes of 38 and 58 patients with chloasma after treatment with Luhui Zhenzhu capsules, respectively, and found that the total effective rate reached 71.05% and 94.83%, respectively (Zhang X. L. et al., 2014; Wei and Feng, 2014).

In terms of eczema, 60 patients with chronic eczema were randomly divided into the treatment group and control group. The treatment group was given compound glycyrrhizin tablets and pearl powder capsules, whereas the control group was only given compound glycyrrhizin tablets. The results showed that the total effective rate of the treatment group was 100%, whereas that of the control group was only 86.67%. The levels of related indicators, interleukin- (IL-) 2, IL-6, and C-reactive protein, also showed a more obvious decrease in the treatment group, suggesting that pearl powder capsules have the effect of promoting the healing of eczema (Pu Y. H. et al., 2018).

In terms of burns and scalds, 150 patients with burns and scalds were selected as experimental subjects and divided into the treatment group and control group. The treatment group was treated with Zhenzhu pulvis and routine nursing care, whereas the control group only received routine nursing care. The results showed that the pain sensation and healing time in the treatment group were milder and shorter than those in the control group, and the number of dressing changes was lesser (Long, 2014).

Regarding skin ulcers, after treatment of 16 skin ulcer patients with pearl powder and Kangfuxin solution, the total effective rate reached 100% (Jiang, 2013). In addition, Zhenzhu pulvis, a compound preparation of pearls, was used to treat 100 patients with chronic skin ulcers, and all patients were cured (Zhu, 2007).

In acne, Xiao et al. (2015) treated 30 acne patients with enzymatic hydrolyzed water-soluble pearl powder capsules and showed that the cure rate was 73.3% after 3 months, which was slightly higher than that 70% of minocycline (Xiao et al., 2015).

### 6.6 Other Diseases

Pearls are equally effective in treating cervical erosions, lateral perineal incision infections, and perianal infections.

Clinically, 94 patients with cervical erosions were divided into the treatment and control groups. The treatment group was treated with pearl powder, and the total effective rate reached 97%. However, the total effective rate of the control group treated with chlorhexidine was only 70%, which was far less than that of the treatment group, indicating that pearl powder is better for the treatment of cervical erosions (Tang et al., 1992).

Lateral perineal incision infection is a common incision complication in the postpartum period. Zhao and Yu (2014) divided 47 maternal patients with lateral perineal incision infections into two groups and treated them with pearl powder and 0.1% rivanol gauze, respectively (Zhao and Yu, 2014). The results showed that the pain relief condition was more obvious, and the recovery rate reached 80% in the patients treated with pearl powder, which was much higher than that of patients treated with 0.1% rivanol.

Saimeian pulvis and pearl powder can effectively treat perianal infections during the onset cycle of acute leukemia. Sixty patients were randomly divided into two groups, and the results showed that the number of effective cases in the treatment group (Saimeian pulvis + pearl powder) was greater than that in the control group (silver sulfadiazine cream), indicating that pearl powder combined with Saimeian pulvis has a better effect on perianal infection of acute leukemia (Chen, 2015).

## 7 Discussion

Pearls have a long history of medicinal use in China as a natural mineral medicine, and modern pharmacology has similarly demonstrated the point of view in ancient books regarding the medicinal effects of pearls (i.e., it can tranquilize and quiet the spirit, improve eyesight and remove nebula, detoxify and promote granulation, moisturize the skin, and remove speckle). However, the exertion of these effects is inseparable from the chemical constituents in pearls (Mo et al., 2015). Amongst these chemical constituents, few active constituents were well defined; however, it is not difficult to speculate on the roles that these constituents play because most of the calcium-like substances, trace elements, and amino acids in pearls are essential constituents of the human body, and many studies have confirmed their value in humans. For example, calcium can promote bone growth and regeneration and may become a bone substitute material (Zhan, 2010; Li et al., 2015). Trace elements such as Se, Zn, Mn, Ge, and Fe can scavenge free radicals in the human body and extend the life of the body; they can treat cardiovascular diseases, regulate the nervous system, resist tumors, and improve anemia symptoms, amongst others (Dong et al., 2011; Li et al., 2015). Amino acids are also essential organic constituents in the human body, maintaining human metabolism and playing other key roles (Liu and Tian, 2010; He et al., 2016). These chemical constituents alone or in conjunction with other constituents achieve the desired physiological function. Therefore, the identification of specific constituents in pearls has also become the cornerstone of subsequent pharmacodynamic studies. Secondly, these specific constituents often have multiple uses for one constituent or one use for multiple constituents, suggesting that we should focus on mining multiple constituents rather than just one constituent in efficacy research.

Amongst the numerous pharmacological activities of pearls, there are many studies on the protection of bone tissue and antioxidant effect, indicating that pearls have good application prospects in these two aspects. Amongst them, the protection of bone tissue is not recorded in ancient books previously, which is an innovative discovery; the anti-oxidation effect is another in-depth study of the moisturizing and freckle-removing effect in the ancient books. This not only shows that pearls have great potential development value, but also suggests the importance of ancient books in medical research. In addition, pearls have been applied to a variety of compound preparations, and under a certain efficacy, these compound preparations also show the same effect as when used with pearls alone. However, due to the large number of medicinal materials contained in compound preparations, the role of pearls in compound preparations is unclear, and the possible effect may be weakened or synergistically enhanced. It is also possible to stimulate another effect, so further experiments are needed for exploration and confirmation. In the current pharmacological experiments on pearls, there is a lack of research on their active constituents, which have also become a breakthrough in future experiments. For example, the research on pearl extraction can be strengthened, and the extraction methods and conditions can be explored to obtain the best extracts. Then, the main constituents can be roughly determined by the extraction method; the extraction method can also be combined with related technologies, such as ICP-MS and LC-MS, to analyze the types of amino acids of the protein and trace elements in the pearl extracts, so as to clarify the active constituents. As most of the results of pharmacological studies were obtained by animal models and their effectiveness could not be fully demonstrated, more clinical trials with confirmatory results are needed (Jiang et al., 2016).

So far, pearls have been clinically applied in the treatment of convulsions, epilepsy, palpitations, insomnia, eye disease, ulcer disease, skin disease, skin injury, and some gynecological diseases, all showing a good curative effect. However, some diseases have complex clinical syndromes, and it is difficult to obtain the desired effect with a single medicine. Thus, pearls are often used in combination with other medicines to treat diseases and often receive satisfactory results. At present, the compound preparations of pearls are also used clinically and have a good curative effect, but their mechanism of action is still unclear. Further research is needed. Although most clinical trials have a small sample size and lack multi-center comprehensive comparative research and unified standards, they still have a certain reference value.

*The Pharmacopoeia of the People’s Republic of China* (Committee, 2020) stipulates that pearls have heavy metals and harmful elements, indicating that their use has certain safety hazards. However, almost all toxicological experimental studies of pearls indicate that they are safe and reliable to use. Although pearls with a slightly higher concentration have certain cytotoxicity, they are within the safety range of the national standard, with a grade of 1, and meet the standards for biomaterial applications in China. However, the number and scope are still insufficient, so more toxicological experiments still need to be carried out to make them more comprehensive and authoritative, laying the foundation for formulating standardized safety standards.

In addition, there is no literature report on the pharmacokinetics of pearls. The absorption, distribution, metabolism, and excretion of pearls in the body after their administration, as well as the changes in blood drug concentrations over time, are unclear. However, some pharmacokinetic studies of pearl preparations, such as Qishiwei Zhenzhu pills (Li and Suo, 2003) and Angong Niuhuang pills (Li et al., 2019), are not enough to replace the pharmacokinetic study of pearls. Therefore, future experiments can also be considered from this aspect to provide a basis for elucidating the pharmacological effects and the design of clinical trials (Wang, 2015). In terms of absorption, the utilization of pearls is not high, so they often enter the human body in the form of powder, which greatly increases the contact area between the medicine and the body, especially nano-pearl powder (Chen L. et al., 2019). On this basis, enzymatic hydrolyzed pearl oral liquid was also derived, and its absorbency became higher due to its solubility (Fan, 2000). Thus, the development of pearls absorption form can be regarded as a research strategy and prospect.

Tibetan medicine is an ethnic medicine that uses the most pearls in addition to traditional Chinese medicine in China, and pearls are used in many Tibetan prescription preparations, such as Qishiwei Zhenzhu pills, Ershiwuwei Zhenzhu pills, and Renqing Changjue. The reasons that Tibetan medicine use pearls can be attributed to two points: one is that pearls are one of the seven treasures of Tibetan Buddhism and have an important position in Tibetan culture (Cao, 2018); the second one is that pearls are a symbol of Tibetan people identity since ancient times, thereby gaining a great deal of attention from the Tibetan people. In addition, from a modern point of view, pearls are mainly produced in Guangxi, Jiangsu, Zhejiang, Anhui, Hunan, Jiangxi, and other places in China, but they are hardly produced in Tibetan areas. Now, convenient transportation has allowed people in Tibetan areas to easily obtain pearls, so the status of pearls in Tibetan areas is not as good as before. However, its medicinal value has been solidified in Tibetan medicine, so pearls are used in a wider range of medicine.

Of course, there will be differences between pearls due to the different production areas. For example, Chinese and Japanese pearls belong to the same variety and are cultivated by *Pteria martensii* Dunke. The difference between the two is mainly reflected in their size, luster, and color; each type has its own merits. However, there is a saying that west pearls are not as good as east pearls, and east pearls are not as good as south pearls (Nan Zhu). South pearls (Nan Zhu) are Chinese seawater pearls, which shows that Chinese pearls have a high reputation around the world.

More trace elements and amino acids in pearls have been studied, whereas other constituents are less studied. The exploration and development of other constituents should be intensified to improve their quality standards and basic research on pharmacodynamic substances. In addition, the pharmacological effects of pearls are numerous and extensive, but there is a lack of research on their mechanism of action. In the future, we should focus on the mechanism research at the gene and molecular levels so that pearls can be better applied in practice. Toxicology research has also come to the fore. The limited clinical trials are not perfect in quality, but they still have a certain reference value. More scientific and representative clinical trials are needed in the future. At present, pearls have been used in many different fields, such as medicine (Cao et al., 1996), cosmetics (Li, 2002), health care (Zhang et al., 2002), and clothing (Hu and Wu, 2020), and more fields are still under development. The value of pearls in medical treatment is particularly significant and needs more attention and extensive research.

## 8 Conclusion

This study comprehensively expounds on the medicinal history of pearls in China and their chemical composition, pharmacology, toxicology, and clinical application for the first time. As a natural mineral medicine, the research on the chemical constituents of pearls has certain limitations; the specific effective constituents are unclear. Most of them use extracts, such as water extraction, acidolysis, and enzymatic hydrolysis. Amongst them, the effect of protein extracts is better, but often a single extraction will lead to the loss of many effective constituents. Therefore, it is often necessary to use a variety of extracts together to make the curative effect better. The specific active constituents need to be further studied. As a new research method, proteomics can be used to explore the specific active constituents of pearls containing protein. Most of the pharmacological effects of pearls have been developed, but most of them live in the superficial part, and the mechanism research has a long way to go. Toxicological studies have confirmed its safety. In terms of clinical application, pearls are mostly used combined with other drugs to treat diseases. The cases of pearls alone to treat diseases are limited, which may lead to the inability to substantively prove the effectiveness of pearls, but these clinical trials still have a certain reference significance. In a word, pearls have great potential development value, and its medicinal value should be well known by more people. In the future, we should focus on the development of pearls taking forms and mechanisms: one is to promote the discovery of more active constituents in pearls, and the other is to lay a foundation for its clinical application.
